# Adaptive-like CAR-iPSC-CD4⁺ T cells outperform CD8⁺ counterparts in sustained ALL control

**DOI:** 10.1186/s41232-025-00402-4

**Published:** 2026-01-03

**Authors:** Qingyi Guo, Chaoqi Zhang, Bo Wang, Shoichi Iriguchi, Akihiro Ishikawa, Atsutaka Minagawa, Tomoko Ishii, Yohei Kawai, Shin Kaneko

**Affiliations:** 1 Shin Kaneko Laboratory, Department of Cell Growth and Differentiation, Center for iPS Cell Research and Application (CiRA), Kyoto University, Kyoto, Japan; 2 Shinobi Therapeutics Inc., South San Francisco, CA USA; 3 Shinobi Therapeutics Inc., Kyoto, Japan; 4 Department of Cancer Immunotherapy and Immunology, Transborder Medical Research Center, University of Tsukuba, Tsukuba, Ibaraki 305-8575 Japan; 5 Department of Cancer Immunotherapy and Immunology, Institute of Medicine, University of Tsukuba, Tsukuba, Ibaraki 305-8575 Japan

**Keywords:** iPS cell, Type 1 CD4^+^ T cell, Immunotherapy, T cell exhaustion, Helper function

## Abstract

**Background:**

Induced pluripotent stem cell (iPSC)–derived T cells offer a renewable source for off-the-shelf immunotherapy. With the advent of the artificial thymic organoid (ATO) method, the in vitro differentiation of CD4^+^ T cells from iPSCs has also become feasible. CD4⁺ T cells have shown superior longevity, resistance to exhaustion, and helper functions in primary settings, but whether iPSC-derived CD4⁺ T cells retain these features remains unclear.

**Methods:**

In this study, CD4⁺ T cells were differentiated from human iPSCs using the ATO system. Primary T cells served as controls to evaluate the phenotypic and activation features of iPSC-derived CD4⁺ and CD8⁺ T cells. To assess antitumor function, we generated CD19-BBζ CAR-iPSC-T cells and employed a hematologic malignancy model using NALM6 acute lymphoblastic leukemia (ALL) cells. Both short-term and long-term cytotoxicity assays were conducted to compare iPSC-derived CD4⁺ and CD8⁺ T cells in terms of killing efficiency, cytokine secretion, persistence, exhaustion phenotype, and proliferative capacity. The helper function of iPSC-derived CD4⁺ T cells toward CD8⁺ T cells was further evaluated by Ki-67 staining and proliferation assays. Statistical analyses were performed using GraphPad Prism.

**Results:**

Our study demonstrated that iPSC-derived CD4⁺ T cells exhibited both helper- and cytotoxic-like features. Compared with iPSC-derived CD8⁺ T cells or CD4⁺/CD8⁺ mixtures, iPSC-derived CD4⁺ T cells showed superior proliferation, cytokine secretion, and sustained cytotoxicity following CAR transduction. They also promoted the expansion of iPSC-derived CD8⁺ T cells and displayed helper-like functions with increased resistance to exhaustion.

**Conclusions:**

Although not identical to primary CD4⁺ T cells, iPSC-derived CD4⁺ T cells recapitulated key functional advantages, especially sustained antitumor activity, supporting their value as a renewable, off-the-shelf source for next-generation CAR-T therapies.

**Supplementary Information:**

The online version contains supplementary material available at 10.1186/s41232-025-00402-4.

## Background

Induced pluripotent stem cells (iPSCs) have gained increasing attention in recent years as a promising source for T cell-based immunotherapies, owing to their unlimited self-renewal capacity and potential to differentiate into various functional cell types. iPSC-derived T (iPSC-T) cells are amenable to genetic manipulation, making them highly suitable for chimeric antigen receptor (CAR)-T and T cell receptor (TCR)-T platforms. These features provide a versatile foundation for cancer immunotherapy [1–3]. However, iPSC-T cells have shown limited persistence in vivo, posing a major challenge for their clinical application. Several strategies—such as gene modification, cytokine armoring, and optimized expansion protocols—have significantly improved the in vivo persistence of iPSC-T cells compared to earlier iterations [4–8]. To further enhance the efficacy of iPSC-T cell therapies, we turned our attention to CD4^+^ T cells.

To date, the majority of iPSC-T cell studies have focused on the CD8⁺ subset, traditionally viewed as the primary cytotoxic effectors in antitumor responses [3]. In contrast, CD4⁺ T cells have long been considered as auxiliary “helper” cells [9]. However, this dichotomy has been increasingly challenged by recent studies, which demonstrate that CD4⁺ T cells can independently exert potent cytotoxic effects mediated by CAR or TCR [10–14]. Primary CD4⁺ T cells have gained attention due to their strong in vivo persistence, ability to support CD8⁺ T cell function, and capacity to activate innate immune components [12, 15–20]. Notably, several studies have shown that primary CD4⁺ T cells are more resistant to exhaustion and can persist for decades in patients, contributing to long-term survival and durable remission [21, 22]. These findings underscore the emerging significance of CD4⁺ T cells in cancer immunotherapy.

Despite this, iPSC-derived CD4⁺ T cells (iCD4⁺ T) remain poorly characterized. Historically, technical limitations in in vitro differentiation systems hindered the generation of CD4⁺ T cells from pluripotent stem cells, favoring the development of CD8⁺ T cells. In 2017, C. S. Seet, et al. reported a major advance using mouse stromal cell–based artificial thymic organoids (ATOs) to support the differentiation of PSCs into CD4⁺ T cells with adaptive-like features and TCRαβ chain [23]. This was later followed by the development of serum and feeder-free systems, which further reduced xenogeneic antigens and improved CD4⁺ T cell yields [24]. However, functional characterization of iCD4⁺ T cells has been limited. Functional investigation of ATO-generated T cells primarily focused on the CD8⁺ subset [25–27], while subsequent studies explored only narrow aspects of iCD4⁺ T cells, particularly their potential to differentiate into regulatory T cells (Tregs) [23, 24, 26, 28]. These studies demonstrated that iCD4⁺ T cells can be induced into iTregs with immunosuppressive capacity. Yet, this alone is insufficient given the well-established plasticity of CD4⁺ T cells, which can differentiate into a wide range of phenotypes [29, 30].

In particular, pro-inflammatory and cytotoxic type 1 CD4⁺ T cells play crucial roles in antitumor immunity [15, 16]. Therefore, the functional and therapeutic potential of adaptive-like iCD4⁺ T cells remains largely unexplored. Whether iCD4⁺ T cells can have functional potency as their primary counterparts, especially in adaptive cell therapy, is still an open question. Moreover, the phenotypical and functional differences between iPSC-derived and primary CD4⁺ T cells remain largely unknown. To address these gaps, we investigated the cytotoxic potential of ATO-generated adaptive-like iCD4⁺ T cells. Leveraging a CD19-BBζ CAR model targeting hematologic malignancies, we evaluated the direct cytolytic activity, durability of antitumor responses, and resistance to repeated antigen stimulation of iPSC-derived CD4^+^ T cells. Additionally, we explored the functional crosstalk between iPSC-derived CD4⁺ and CD8⁺ T cells and evaluated whether their combined use could enhance therapeutic efficacy beyond what is achievable with either subset alone. Our findings reveal that iCD4⁺ T cells not only exhibit canonical helper T cell features but also possess robust proliferative capacity and direct cytotoxic function. Notably, these cells display sustained antitumor efficacy independent of iPSC-derived CD8⁺ T cells (iCD8^+^ T) and outperformed their iCD8⁺ counterparts. Together, these results highlight iCD4⁺ T cells as a potent and underutilized cell population with strong potential for PSC-based next-generation T cell-based immunotherapies.

## Methods

### Cells

#### PBMC

Peripheral blood mononuclear cells (PBMCs) were obtained from healthy donors who provided written informed consent.

#### iPSC

The T-iPSC lines used in this study included TKT3V1-7 [31] and b3a2#9 [32], which were reprogrammed from peripheral blood T cells using Sendai virus vectors. Another iPSC line, FFI01s04, is an HLA-homozygous line established at CiRA and distributed with informed consent and ethical approval by the CiRA Ethical Review Board. The FFI01s04-b3a2 line was generated by transducing the TCR derived from b3a2#9 into FFI01s04 via lentiviral infection, as previously described [33]. All iPSC lines were maintained in StemFit AK02N medium (Ajinomoto, Japan) on iMatrix-511 (Matrixome, Japan) as previously described [34]. All studies involving human samples were approved by the Kyoto University School of Medicine Ethics Committee (approval no. G590).

#### MS5/DL4

The MS5 mouse stromal cell line was obtained from the DSMZ cell bank and transduced with human Delta-like ligand 4 (DLL4) using lentiviral vectors, as previously described [28]. MS5/DL4 cells were cultured in αMEM (Thermo Fisher Scientific) supplemented with 10% fetal bovine serum (FBS, HyClone) and 1 × L-glutamine–penicillin–streptomycin solution (PSG, Thermo Fisher Scientific).

#### NALM6 and K562

NALM6 and K562 cell lines were purchased from the RIKEN Cell Bank and JCRB Cell Bank, respectively. Cells were maintained in RPMI-1640 medium supplemented with 10% FBS.

All the cells were cultured at 37 °C in a humidified incubator with 5% CO₂. Mycoplasma contamination was routinely tested and confirmed to be negative.

### Flow cytometry and antibodies

All flow cytometry staining was performed in PBS supplemented with 2% FBS (FACS buffer) on ice for 30 min. Intracellular staining was carried out using Fixation Buffer (BioLegend, Cat. No. 420801) and Intracellular Staining Perm Wash Buffer (BioLegend, Cat. No. 421002), following the manufacturer’s instructions. For intracellular cytokine detection, T cells were pre-stimulated with 25 ng/mL PMA and 1 μg/mL ionomycin for 4 h in the presence of 1 × monensin (BioLegend) to inhibit cytokine secretion. Cell viability was assessed using propidium iodide (PI) for non-fixed samples and the LIVE/DEAD™ Fixable Aqua Dead Cell Stain Kit (405 nm excitation; Thermo Fisher Scientific) for fixed cells. Flow cytometry and cell sorting were performed on a BD FACSymphony™ S6, FACS Aria II, or Aria Fusion instrument (BD Biosciences, San Jose, CA, USA) using FACSDiva software (v8.0.1). Data were analyzed with FlowJo software (Tree Star Inc.).

Antibodies used: From BioLegend:CD1a (HI149), CD3 (UCHT1), CD4 (OKT4), CD5 (UCHT2), CD7 (M-T701), CD8β (SIDI8BEE), CD25 (BC96), CD27 (O323), CD28 (CD28.2), CD45RA (HI100), CD45RO (UCHL1), CD56 (HCD56), CD69 (FN50), CD154/CD40L (24–31), CCR7 (G043H7), CCR4 (L291H4), CXCR3 (G025H7), CD336/NKp44 (P44-8), CD337/NKp30 (P30-15), CD314/NKG2D (1D11), CD253/TRAIL (RIK-2), CD355/CRTAM (Cr24.1), CD178/FasL (NOK-1), ThPOK (ZFP-67), CD278/ICOS (29E.2A3), CD137/4-1BB (4B4-1), CD279/PD-1 (EH12.2H7), CD366/TIM-3 (F38-2E2), CD152/CTLA-4 (L3D10), EGFR (AY13), TCRαβ (IP26), IL-2 (MQ1-17H12), IL-4 (MP4-25D2), IL-21 (4BG1), TNF-α (MAb11), Granzyme B (GB11). From BD Biosciences: CD62L (SK11). From Miltenyi Biotec: IFN-γ (REA600). From Invitrogen: TIGIT (MBSA43), CD223/LAG-3 (3DS223H). From BD Pharmingen: Ki-67 (B56).

### Generation of HPCs by EB method

The protocol for differentiating iPSCs into hematopoietic progenitor cells (HPCs) was based on a previous paper, with slight modifications [34]. Briefly, the induction of embryoid body (EB) starts with placing 3 × 10^5^ iPSCs in 6-well ultra-low attachment plates for 24 h in StemFit AK02N medium supplemented with 10 μM Y-27632 (FujiFilm Wako) and 10 μM CHIR99021 (Tocris Bioscience). Afterward, the medium was changed to EB basal medium containing 50 ng/mL rhVEGF (R&D Systems), 50 ng/mL rhbFGF (FujiFilm Wako), and 50 ng/mL rhBMP-4 (R&D Systems). The EB basal medium consisted of StemPro-34 (Thermo Fisher Scientific) supplemented with 1 × PSG, 1 × GlutaMAX (Thermo Fisher Scientific), 50 μg/mL ascorbic acid-2-phosphate (Sigma), 4 × 10⁻^4^ M monothioglycerol (MTG; Nacalai), and 1 × ITS-G (Thermo Fisher Scientific). After 24 h, 6 μM SB431542 (FujiFilm Wako) was added to the culture. On day 4, the medium was replaced with EB basal medium containing 50 ng/mL rhVEGF, 50 ng/mL rhbFGF, and 50 ng/mL rhSCF (R&D Systems). On day 6, 10 ng/mL rhFLT3L (PeproTech) and 30 ng/mL rhTPO (PeproTech) were added. Cultures were maintained in an incubator with 5% CO₂, 5% O₂, and 90% N₂ for the first 6 days and then transferred to a standard 5% CO₂ incubator from day 7 to day 14. On day 14, floating cells were collected as HPCs.

### T cell differentiation via ATO method

T cell differentiation was performed using the ATO method, as previously described [23, 28]. HPCs generated by the EB method were mixed with MS5/DL4 feeder cells at a ratio of 1:5 to 1:10. After centrifugation at 300 × *g* for 5 min, the supernatant was removed, and the cell pellet was transferred onto 0.4-μm Millicell Transwell inserts (Merck Millipore, Cat. PICM0RG50). The inserts were then placed in 6-well plates (TPP) containing 1.3 mL of T cell induction medium per well. Medium was completely replaced every 3 days. After 7–9 weeks of culture, mature iCD4⁺ and iCD8⁺ T cells were harvested via flow cytometric sorting. The T cell induction medium consisted of α-MEM (Thermo Fisher Scientific) supplemented with 1 × GlutaMAX, 1 × PSG, 4% B27 supplement (Thermo Fisher Scientific), 50 μg/mL ascorbic acid-2-phosphate, 5 ng/mL rhIL-7 (PeproTech), 10 ng/mL rhSCF, 5 ng/mL rhFLT3L, 30 nM rhSDF-1α (PeproTech), and 15 μM SB203580 (Tocris Bioscience).

### T cell activation and expansion

Sorted CD4⁺ and CD8⁺ T cells were activated by co-culturing with Dynabeads Human T-Activator CD3/CD28 (Thermo Fisher Scientific) at a 1:1 bead-to-cell ratio in 200 μL of T cell expansion medium in 96-well U-bottom plates. For iPSC-derived T cells, additional cytokines—20 ng/mL rhIL-21 (PeproTech), 50 ng/mL rhIL-12 (Merck), and 50 ng/mL rhIL-18 (MBL)—were added at the time of activation. No supplemental cytokines were added for primary T cells. After 2 days, Dynabeads were removed, and cells were transferred to 96-well flat-bottom plates. Cultures were expanded, and media volumes adjusted as needed based on cell proliferation. The T cell expansion medium consisted of α-MEM supplemented with 15% FBS, 1 × GlutaMAX, 1 × PSG, 1 × ITS, 5 ng/mL rhIL-7, 5 ng/mL rhIL-15 (PeproTech), and 50 μg/mL ascorbic acid-2-phosphate.

### In vitro* cytotoxicity assays*

#### Generation of CAR-T cells

The construction of CD19-BBζ CAR and generation of CAR-T cells were performed as previously described [34]. The CAR comprises a single-chain variable fragment (scFv) targeting CD19 (clone FMC63), a CD8 hinge and transmembrane domain, the 4-1BB co-stimulatory domain, and the CD3ζ signaling domain, and was introduced into T cells using the pMYs γ-retroviral system. On day 3 after Dynabeads stimulation, T cells were transferred to Retronectin-coated (TAKARA) 96-well flat-bottom plates and spin-infected with retroviral supernatant at 1000 × *g*, 32 °C for 60 min. The number of T cells was adjusted to approximately 50% confluency for transduction. EGFR-expressing CAR-T cells were subsequently enriched by flow cytometric sorting.

#### Short-term cytotoxicity assay

To assess target cell killing efficiency, NALM6 and K562 cells were transduced with a retrovirus-mediated gene delivery system to express Firefly luciferase (FLuc) and GFP. For short-term cytotoxicity assays, 1 × 10^4^ target cells were co-cultured with CAR-T cells at varying E:T ratios. After 3 h of incubation, D-luciferin substrate (100 μg/mL) was added, and luminescence was measured using a VICTOR Nivo multimode plate reader (PerkinElmer). Killing efficiency was calculated using the formula:

$$\%\ \mathrm{Killing}\:=\:\lbrack(\mathrm{Luminance}_{\;\mathrm{Target}\;\mathrm{only}}\:-\:\mathrm{Luminance}_{\;\mathrm{co}-\mathrm{culture}})/(\mathrm{Luminance}_{\;\mathrm{Target}\;\mathrm{only}}\:-\:\mathrm{Luminance}_{\;\mathrm{medium}\;\mathrm{only}})\rbrack\:\times\:100.$$

#### Multi-round tumor challenge assay

For serial killing assays, 1 × 10^4^ CAR-T cells were co-cultured with 5 × 10^4^ NALM6-FLuc cells. Killing efficiency was assessed every 3 days, and fresh 5 × 10^4^ NALM6-FLuc cells were added at each time point until the killing rate dropped below 80%, at which point no additional target cells were added and that cycle was defined as the final round. At the fourth round of co-culture, the absolute number of T cells was determined by flow cytometry using CountBright™ Absolute Counting Beads (Thermo Fisher Scientific).

### Cytokine production

Supernatants from the first round of the multi-round tumor challenge assay were collected and analyzed for cytokine concentration using the BD™ Cytometric Bead Array (CBA) Human Th1/Th2/Th17 Kit (BD Biosciences), according to the manufacturer’s instructions.

### Quantitative real-time PCR

T cells on day 9 of initial expansion were washed with PBS and lysed for total RNA extraction using the RNeasy Micro Kit (QIAGEN) according to the manufacturer’s protocol. Complementary DNA (cDNA) was synthesized using the PrimeScript™ 1st Strand cDNA Synthesis Kit (TAKARA). Real-time quantitative PCR was performed using pre-designed gene-specific TaqMan Gene Expression Assay probes and 2 × TaqMan Fast Advanced Master Mix (Applied Biosystems). The following probes were used: *ACTB* (Hs01060665_g1), *TCF7* (Hs00175273_m1), *TOX* (Hs01049519_m1), *TBX21* (Hs00894392_m1), and *PRDM1* (Hs00153357_m1). Reactions were run on a QuantStudio™ 3 Real-Time PCR System (Applied Biosystems). Relative gene expression was calculated using the ΔΔCt method: ΔCt = Ct_target_ − Ct_ACTB_; ΔΔCt = ΔCt_CD8_ − ΔCt_CD4_; Relative expression = 2^(−ΔΔCt)^.

### Enrichment analysis

RNA-seq data from the GEO database (GSE116015) were used to perform enrichment analysis on ATO-derived ESC-CD4^+^ and ESC-CD8^+^ T cells. Differential expression analysis was conducted using GEO2R, an online tool provided by the National Center for Biotechnology Information (NCBI) (https://www.ncbi.nlm.nih.gov/geo/geo2r). The top 250 differentially expressed genes (DEGs) for ESC-CD4^+^ and ESC-CD8^+^ T cells, respectively, were submitted to the Metascape online platform (https://metascape.org) [35] for Gene Ontology (GO) and pathway enrichment analysis.

## Results

### Generation and activation of adaptive-like iCD4^+^ T cells

**Fig. 1:**
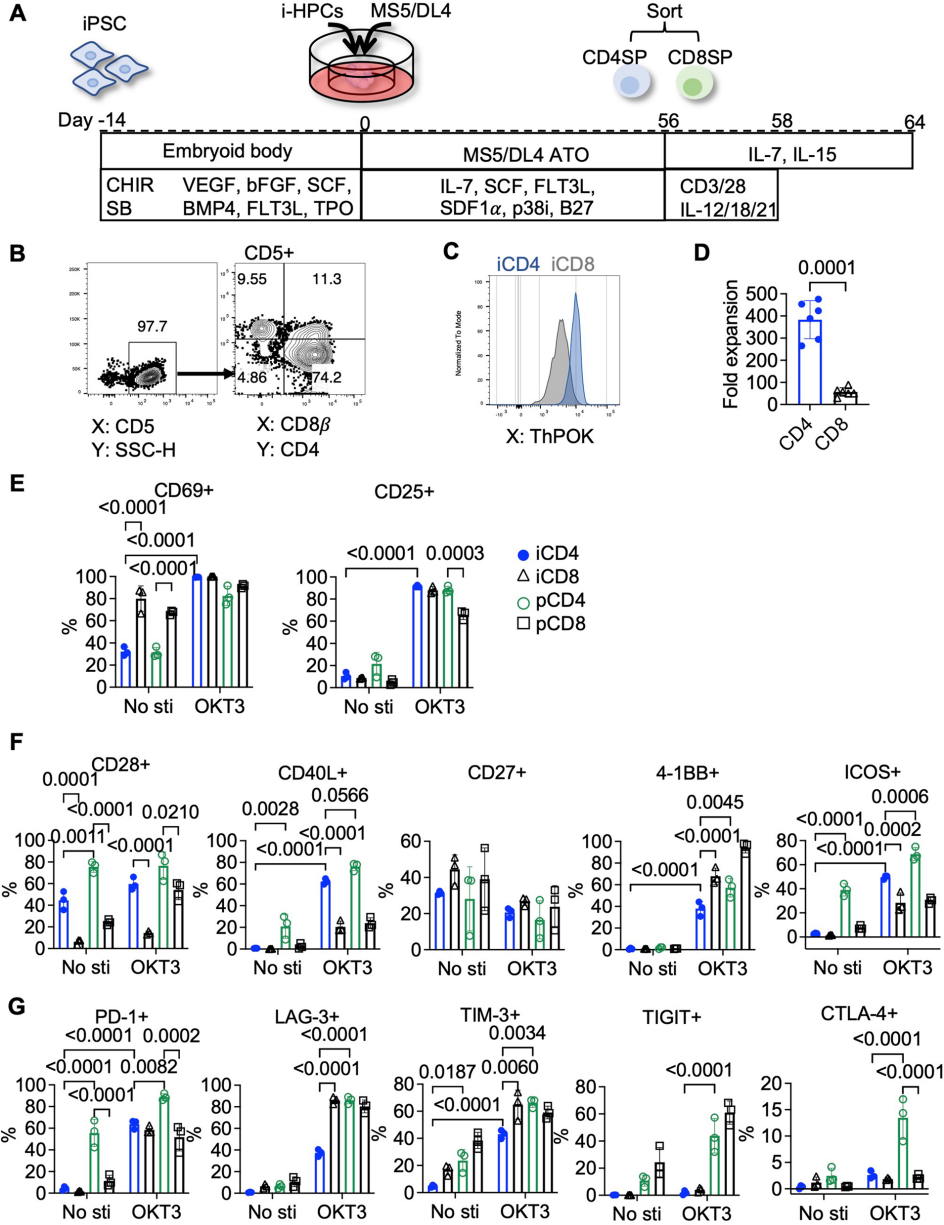
Generation and functional activation of ATO-derived iCD4⁺ T Cells. **A** Schematic overview of the differentiation and expansion protocol for iPSC-derived CD4⁺ and CD8⁺ T cells. iPSCs were first differentiated into hematopoietic progenitor cells (HPCs) via embryoid body (EB) formation, followed by induction into T cells using the artificial thymic organoid (ATO) system, and then subjected to primary in vitro expansion. **B** Representative flow cytometry plots of iPSC-derived T cells harvested from Week8 ATO cultures. **C** Representative flow cytometry plots showing the CD4 lineage-specifying transcription factor ThPOK expression in ATO-derived iCD4⁺ and iCD8⁺ T cells. **D** Fold expansion of iCD4⁺ and iCD8⁺ T cells over 8 days following primary stimulation. *n* = 6; paired *t* test. **E**–**G** On day 14 post-expansion, resting iPSC-derived and primary T cells were either unstimulated or stimulated with plate-bound anti-CD3 antibody (OKT3) for 24 h. Flow cytometric analysis was used to assess expression of activation markers (CD69, CD25) (**E**), co-stimulatory receptors (CD28, CD40L, CD27, 4-1BB, ICOS) (**F**), and inhibitory receptors (PD-1, LAG-3, TIM-3, TIGIT, CTLA-4) (**G**). *n* = 3; statistical analysis used ordinary two-way ANOVA followed by Tukey’s multiple comparisons test with a single pooled variance. Corresponding representative flow cytometry plots are shown in Fig. S3. Note: Experiments were performed using the TKT3V1-7 iPSC line. Data are shown as mean ± SD from independent experiments (*n* values specified in figure legends) unless otherwise indicated

To evaluate the functional potential of iCD4⁺ T cells, we employed the ATO system [23]. Although a previous study has reported that feeder-free protocols with PMA stimulation can generate CD4⁺ T cells from iPSCs, this approach appears to drive a non-physiological maturation trajectory—progressing from CD8 immature single positive cells (SP) to CD4 SP—and yields T cells exhibiting innate lymphoid cell (ILC)-like characteristics [24]. To better emulate canonical αβ T cell development, we therefore utilized the ATO method, which more faithfully mirrors physiological T-cell development. We first generated hematopoietic progenitor cells from iPSCs through embryoid body formation, followed by ATO-based T cell differentiation using mouse stromal cell line MS5 cells overexpressing Delta-like 4 (MS5/DL4), as shown in Fig. 1A [23, 34, 36, 37]. The ATO culture was supplemented with low concentrations of IL-7, SCF, and FLT3L to support T cell differentiation. In addition, SDF1α and the p38 MAPK inhibitor (p38i) SB203580, which have been shown to enhance feeder-free T cell differentiation, were included in the culture medium [34]. After approximately 4 weeks, CD4⁺CD8⁺ double-positive (DP) immature T cells emerged within the ATOs, expressing canonical T lineage markers such as CD7, CD5, and CD1a (Fig. S1A). By weeks 7–9, DP cells spontaneously matured into CD4⁺ and CD8⁺ SP subsets (Fig. 1B, Fig. S1A).

To evaluate how iPSC origin influences T cell differentiation, we compared T cell differentiation using both T-iPSC lines (e.g., TKT3V1-7) with HLA homozygous non-T-iPSC lines (e.g., FFI01s04) [31]. As expected, T-iPSCs—reprogrammed from mature T cells and thus retaining rearranged TCR loci—in contrast, non-T-iPSCs yielded a broader repertoire, including both αβ and γδ T cells (Fig. S1A). To determine whether CD4/CD8 lineage commitment is influenced by the origin of the iPSC or the TCR MHC restriction, we examined the percentage of CD4⁺ and CD8⁺ T cells from four iPSC lines: T-iPSCs (TKT3V1-7, b3a2#9), non-T-iPSCs (FFI01s04), and TCR-transgenic non-T-iPSCs (FF-b3a2) [32, 38]. The b3a2 TCR specifically recognizes MHC class II and was originally derived from a CD4⁺ T cell targeting the BCR-ABL fusion protein in leukemia [38, 39]. Regardless of iPSC origin or TCR specificity, all iPSC lines produced similar CD4^+^ and CD8^+^ T cell ratios, indicating that lineage decision in this system is not dictated by TCR rearrangement or MHC class restriction (Fig. S1B).

By approximately week 8, CD4 SP cells acquired a mature CD4⁺ T cell phenotype, characterized by the expression of the lineage-defining transcription factor ThPOK (Fig. 1C) and the chemokine receptors CCR4 and CCR7. These cells also upregulated naïve-associated markers such as CD45RA and CD28, while downregulating immature T cell marker CD1a (Fig. S1A, Fig. S2A, B) [40, 41]. We then isolated iCD4⁺ and iCD8⁺ T cells and expanded them using Dynabeads Human T-Activator CD3/CD28 in the presence of cytokines known to support iCD8⁺ T cell expansion and type 1 polarization, including IL-7, IL-15, IL-12, IL-18, and IL-21 [5, 29]. Under these conditions, iCD4⁺ T cells derived from TKT3V1-7 displayed robust proliferative capacity, surpassing that of their CD8⁺ counterparts and approaching the expansion levels of primary T cells cultured under clinically relevant IL-7 and IL-15 conditions (Fig. 1D; Fig. S2C). A similar proliferative advantage was also observed in CD4⁺ T cells differentiated from the non-T iPSC line FFI01s04 (Fig. S2D). In contrast, primary CD4^+^ and CD8^+^ T cells showed comparable expansion efficiencies during initial expansion (Fig. S2C). Notably, iCD4⁺ T cells expressed higher and more sustained levels of the co-stimulatory receptor CD28 compared to iCD8⁺ T cells, which may underlie their superior proliferative capacity (Fig. 1F, Fig. S2A, B).

Upon activation, T cells upregulate a variety of co-stimulatory receptors—including 4-1BB, CD28, and ICOS—as well as inhibitory receptors such as PD-1, LAG-3, and TIM-3, to fine-tune TCR signaling intensity [42]. The transient induction of inhibitory receptors during activation serves as a negative feedback mechanism to prevent overactivation and cytokine storms, and does not necessarily indicate exhaustion [42]. CD4⁺ T cells additionally upregulate the helper ligand CD40L upon activation, enabling them to support other immune cells such as B cells, dendritic cells, and macrophages—functions distinct from those of CD8⁺ T cells [43–45]. To compare activation phenotypes between iCD4⁺ and iCD8⁺ T cells, we stimulated them using two approaches: plate-bound CD3 agonist antibody (OKT3) or PMA/ionomycin, which bypasses TCR by directly activating the MAPK pathway and triggering calcium influx. Primary CD4⁺ and CD8⁺ T cells from three healthy donors were stimulated in parallel as controls. As shown in Fig. 1E–G and Fig. S3, both iCD4⁺ and iCD8⁺ T cells expressed low or undetectable levels of CD25, 4-1BB, ICOS, and inhibitory receptors at resting state. Notably, iCD8⁺ T cells exhibited higher basal expression of CD69 compared to iCD4⁺ T cells. Upon stimulation, both subsets rapidly upregulated CD69 and CD25, accompanied by differential induction of co-stimulatory and inhibitory receptors. iCD4⁺ T cells exhibited several hallmark features consistent with a helper phenotype, closely resembling their primary counterparts: (1) strong upregulation of CD40L upon activation, reinforcing their helper identity; (2) high baseline expression of CD28, which was further elevated following OKT3 stimulation (Fig. 1F); (3) marked upregulation of ICOS from low basal levels upon activation (Fig. 1F). These features are functionally interconnected; for example, CD28 signaling enhances the expression and stability of helper ligands like CD40L and ICOS [46, 47]. Although iCD8⁺ and primary CD8⁺ T cells also upregulated these receptors upon activation, the percentage of positive cells was substantially lower than that observed in CD4⁺ T cells. In contrast, both iPSC-derived and primary CD8^+^ T cells expressed higher levels of CD27 at rest and showed stronger upregulation of 4-1BB upon activation, consistent with previous reports [48–50]. Notably, 4-1BB signaling is preferentially associated with enhancing CD8⁺ T cell effector function [51]. Overall, iPSC-derived CD4⁺ and CD8⁺ T cells recapitulate key subset-specific activation phenotypes observed in primary CD4⁺ and CD8⁺ T cells, reflecting lineage-appropriate functional programming.

In contrast to co-stimulatory receptors, inhibitory receptors were differentially upregulated in iPSC-derived and primary T cells following activation (Fig. 1G). Primary CD4⁺ T cells exhibited markedly higher transient expression of inhibitory receptors such as LAG-3, PD-1, TIM-3, TIGIT, and CTLA-4 compared to their iPSC-derived counterparts. Among iPSC-derived T cells, PD-1, LAG-3, and TIM-3 were notably upregulated upon activation, with iCD8⁺ T cells expressing higher levels of LAG-3 and TIM-3 than iCD4⁺ T cells. However, the expression of TIGIT and CTLA-4 remained nearly undetectable. The overall lower expression of co-inhibitory receptors in iPSC-derived T cells may reflect either a more sustained activation profile or insufficient activation when compared to primary T cells.

### iCD4⁺ T cells exhibit both helper-like and cytotoxic features

**Fig. 2:**
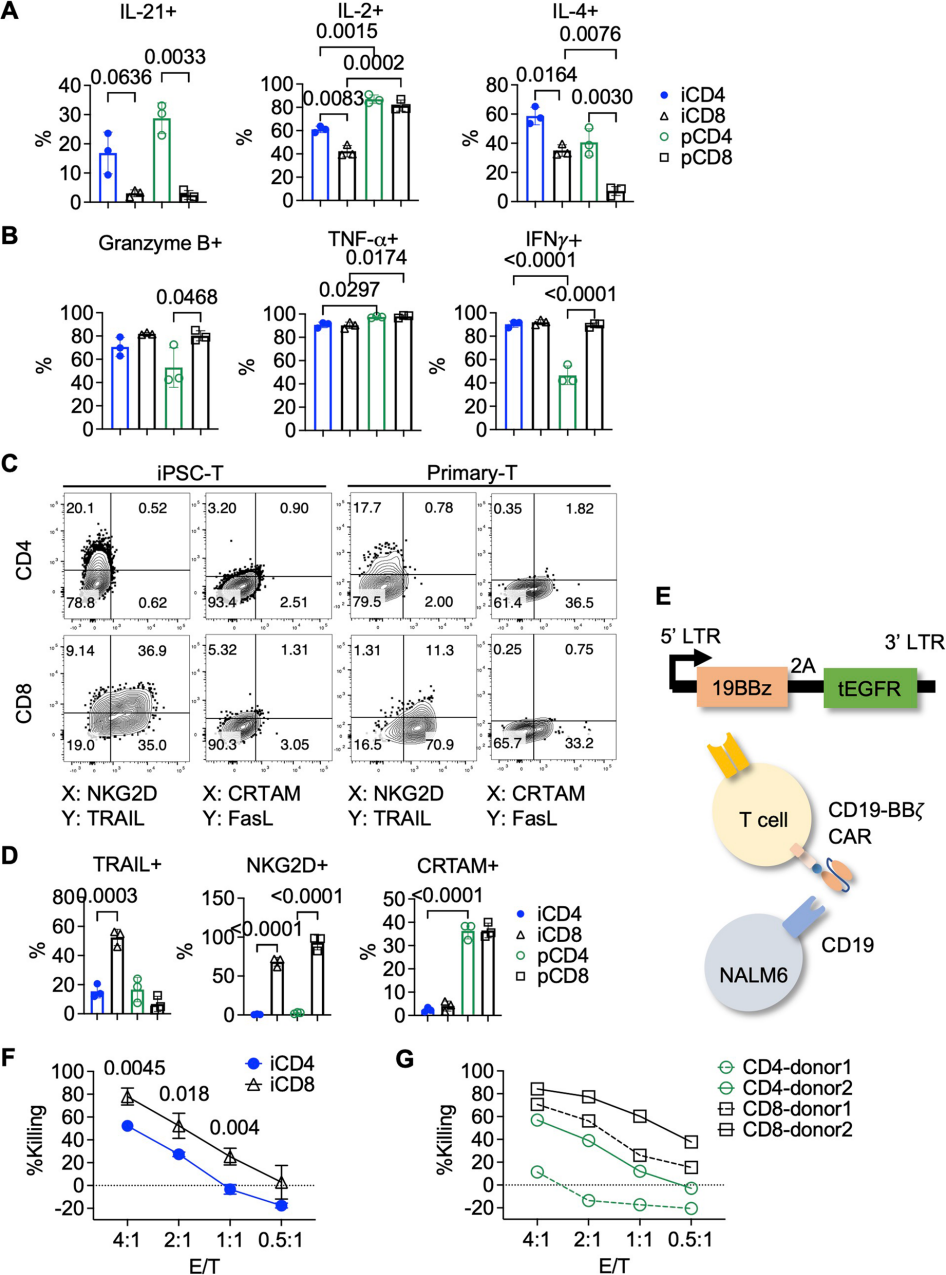
iCD4⁺ T cells exhibit killer-like properties. **A**, **B** Percentage of cells producing helper-associated cytokines (**A**) and cytotoxic-associated cytokines (**B**) after 4 h of PMA/ionomycin stimulation. *n* = 3; RM one-way ANOVA with Tukey’s test. Corresponding representative flow cytometry plots are shown in Fig. S4. **C** Representative flow cytometry plots showing the expression of killer ligands on expanded iPSC-derived and primary CD4⁺ and CD8⁺ T cells. **D** Frequencies of TRAIL⁺, NKG2D⁺, and CRTAM⁺ cells among iPSC-derived and primary CD4⁺ and CD8⁺ T cells. *n* = 3, ordinary one-way ANOVA with Tukey’s multiple comparisons test. **E** Schematic of the CD19-BBζ CAR retroviral construct and CAR-dependent killing of NALM6 cells. The truncated EGFR (tEGFR) is co-expressed as a marker via a 2 A peptide linker. **F** Cytotoxicity of CD19-BBζ CAR-expressing iPSC-derived CD4⁺ and CD8⁺ T cells against NALM6 cells in a 3-h killing assay. *n* = 3; multiple unpaired *t* tests with two-stage step-up method of Benjamini, Krieger, and Yekutieli. **G** Cytotoxic activity of CD19-BBζ CAR-expressing primary CD4⁺ and CD8⁺ T cells from two different donors against NALM6 cells in a 3-h killing assay. Note: Experiments were performed using the TKT3V1-7 iPSC line. Data are shown as mean ± SD from independent experiments (*n* values specified in figure legends) unless otherwise indicated

CD4⁺ T cells exert helper functions primarily through the expression of CD40L and the secretion of cytokines such as IL-21 and IL-2 [52]. We have demonstrated that iCD4⁺ T cells upregulate CD40L upon activation to levels comparable to those of primary CD4⁺ T cells (Fig. 1F). To further evaluate their cytokine-producing capacity, we stimulated iCD4⁺ T cells with PMA/ionomycin and performed intracellular cytokine staining after 4 h. As shown in Fig. 2A and Fig. S4A, the proportion of IL-2⁺ cells among iCD4⁺ T cells (~ 60%) was lower than that in primary CD4⁺ T cells (~ 80%), but significantly higher than in iCD8⁺ T cells (~ 40%). Notably, a subset of iCD4⁺ T cells also produced IL-21—a feature absent in both iPSC- and primary CD8⁺ T cells. In addition, more than half of the iCD4⁺ T cells secreted IL-4, a cytokine associated with type 2 immune responses and B cell proliferation [53]. This observation was confirmed in both T-iPSC line TKT3V1-7 (Fig. 2A, Fig. S4A) and non-T iPSC line FFI01s04 (Fig. S4B, C). Together, these findings demonstrate that iCD4⁺ T cells possess distinct and functional helper properties.

CD4⁺ T cells can acquire cytotoxic features under inflammatory and tumor conditions, including the secretion of granzymes, perforin, and IFN-γ, as well as the surface expression of killer ligands such as CRTAM, FasL, NKG2D, and TRAIL (CD253), which induce apoptosis in target cells [15, 16]. To evaluate the cytotoxic profile of iCD4⁺ T cells, we examined their expression of killer ligands and production of effector molecules. Upon expansion in type 1 cytokine conditions described above, both iCD4⁺ and iCD8⁺ T cells acquired a Th1-biased chemokine receptor profile, characterized by high CXCR3 and low CCR4 expression (Fig. S2F, G). Following PMA/ionomycin stimulation, intracellular cytokine staining revealed that the majority of iCD4⁺ and iCD8⁺ T cells produced granzyme B, IFN-γ, and TNF-α within 4 h (Fig. 2B; Fig. S4A). In parallel, surface staining showed expression of the killer ligand TRAIL on both iPSC- and primary-derived CD4⁺ T cells (Fig. 2C, D). Notably, TRAIL expression was absent prior to initial expansion, suggesting that this cytotoxic-associated feature requires priming (Fig. S2A). Other killer ligands, such as FasL and NKG2D, were not detected on either iPSC- or primary CD4⁺ T cells. Compared to CD4⁺ T cells, both iPSC- and primary-derived CD8⁺ T cells exhibited a higher proportion of granzyme B⁺ cells and strongly expressed NKG2D, consistent with a more canonical cytotoxic phenotype. Interestingly, CRTAM was undetectable on both iCD4⁺ and iCD8⁺ T cells, in contrast to its presence in primary T cells (Fig. 2C, D). Overall, although less potent than iCD8⁺ T cells, iCD4⁺ T cells expanded under type 1 cytokine conditions described above naturally acquired cytotoxic traits, highlighting their dual helper and killer potential.

To evaluate the cytotoxic activity of iCD4⁺ T cells, we transduced them with a second-generation CAR construct comprising a CD19-specific single-chain variable fragment (scFv) fused to 4-1BB and CD3ζ signaling domains. These CAR-iCD4⁺ T cells were co-cultured with the CD19⁺ acute lymphoblastic leukemia cell line NALM6 for a short-term (3-h) killing assay, as shown in Fig. 2E. CAR-transduced primary CD4⁺ and CD8⁺ T cells from different donors served as controls. As shown in Fig. 2F, G, both iPSC-derived and primary CD4⁺ T cells exhibited CAR-mediated cytotoxicity; however, CD8⁺ T cells—whether iPSC-derived or primary—achieved more rapid and efficient clearance of target cells during the brief co-culture period. This superior killing efficiency is consistent with the more prominent cytotoxic phenotype typically observed in CD8⁺ T cells. Taken together, these results indicate that iCD4⁺ T cells possess direct cytolytic activity akin to their primary counterparts, albeit at a lower magnitude, and likely retain concurrent helper functionality.

### iCD4⁺ T cells exhibit a reduced NK-like phenotype relative to iCD8⁺ T cells

**Fig. 3:**
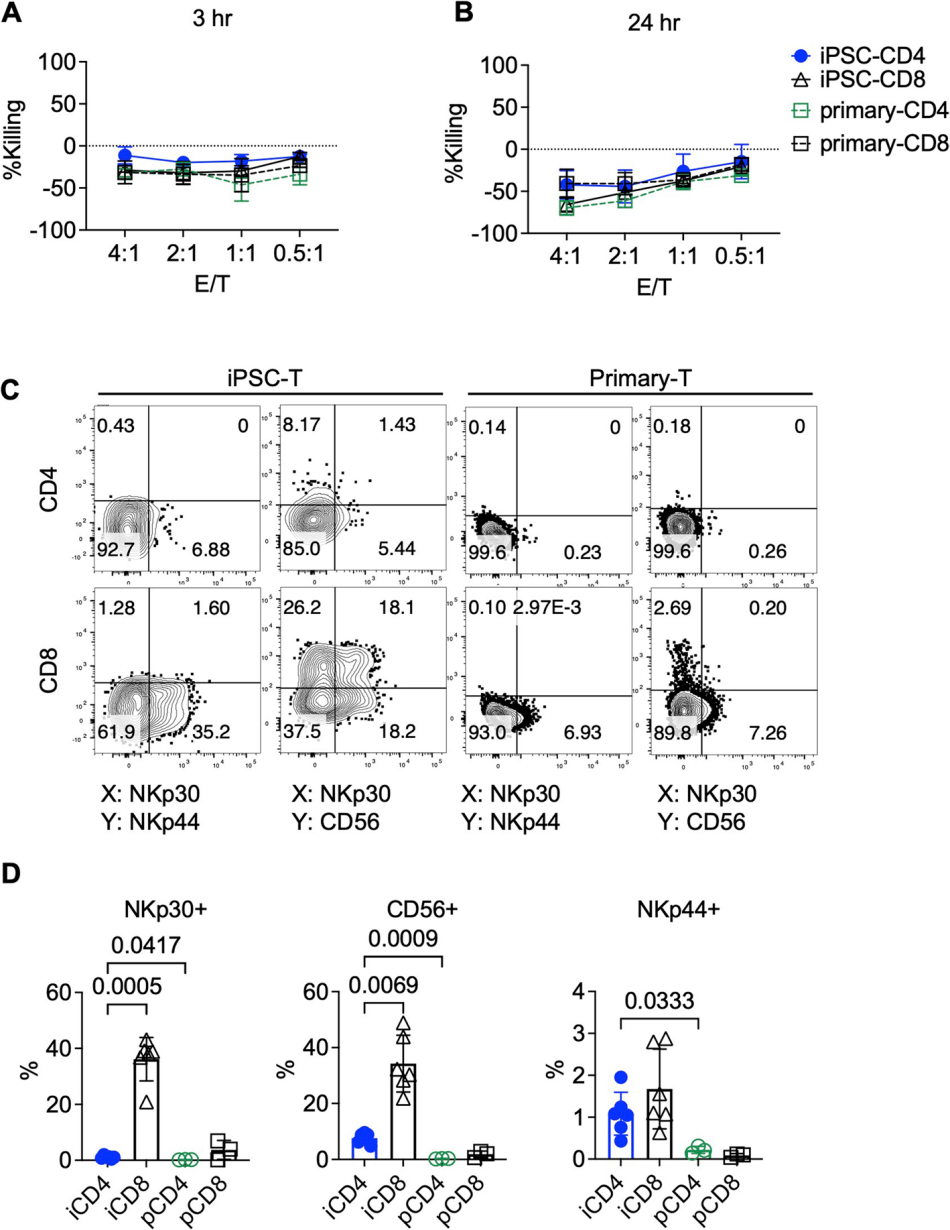
NK-like features of iCD4⁺ and iCD8⁺ T cells. **A**, **B** Cytotoxicity of CAR-expressing primary and iPSC-derived CD4⁺ and CD8⁺ T cells against K562 cells after 3 h (**A**) and 24 h (**B**) of co-culture. *n* = 3. **C** Representative flow cytometry plots showing NK-associated surface marker expression on iPSC-derived and primary CD4⁺ and CD8⁺ T cells after primary expansion. **D** Percentage of NKp30^+^, CD56^+^, and NKp44^+^ populations on iPSC-derived and primary CD4⁺ and CD8⁺ T cells. *n *= 6 for iPSC-derived cells; *n* = 3 for primary cells. Statistical analysis was performed using Brown-Forsythe and Welch ANOVA tests followed by Dunnett’s T3 multiple comparisons test with individual variances computed for each comparison. Note: Experiments were performed using the TKT3V1-7 iPSC line. Data are shown as mean ± SD from independent experiments (*n* values specified in figure legends) unless otherwise indicated

It is well recognized that iPSC-derived T cells can display innate-like characteristics. Under early OP9/DLL1 differentiation protocols, iCD8⁺ T cells were reported to express innate markers such as NKp44 and CD56 and to exert antigen-independent cytotoxic activity [5]. However, excessive innate-like functionality raises concerns about potential off-target effects and in vivo toxicity. The ATO method has been proposed to mitigate such risks by avoiding agonist selection, thereby reducing innate-like bias during differentiation [26]. Previous studies have shown that T cells generated using the ATO method do not kill HLA-deficient K562 cells and exhibit low NKp44 expression, though they still exhibit elevated CD56 expression compared to primary T cells—indicating residual innate-like features [25]. Notably, these prior evaluations focused on bulk iPSC-T cell populations or iCD8⁺ subsets, leaving the innate-like phenotype of ATO-derived iCD4⁺ T cells largely uncharacterized. To address this, we assessed innate cytotoxicity by co-culturing CAR-iCD4⁺ and CAR-iCD8⁺ T cells, as well as primary CAR-T cells, with K562 target cells at various effector-to-target (E:T) ratios (ranging from 4:1 to 0.5:1) for 3 to 24 h. As shown in Fig. 3A, B, none of the T cell populations—regardless of subset, E:T ratio, or co-culture duration—exhibited cytotoxic activity against K562 cells. These results confirm that the cytotoxicity observed against CD19⁺ NALM6 cells was CAR specific, rather than mediated by innate-like mechanisms. We further evaluated the expression of innate markers that are typically absent in primary T cells, including CD56, NKp44, and NKp30 (Fig. 3C, D). Consistent with previous findings [25], iPSC-derived T cells showed no detectable NKp44 expression but did express CD56. In addition, NKp30 expression was observed in ATO-derived iPSC-T cells. Notably, both CD56 and NKp30 were predominantly expressed in a subset of iCD8⁺ T cells, while iCD4⁺ T cells displayed minimal expression of these markers (Fig. 3C, D). Supporting these findings, re-analysis of transcriptomic data from A. Montel-Hagen et al. (GSE116015) revealed that genes associated with NK cell activation were also more highly enriched in ATO-derived ESC-CD8⁺ T cells compared to CD4⁺ counterparts (Fig. S6) [26]. Together, these findings indicate that iCD4⁺ T cells possess fewer innate-like features and more closely resemble conventional adaptive T cells.

### CAR-iCD4⁺ T cells exhibit superior and sustained antitumor activity compared to iCD8⁺ T cells

**Fig. 4:**
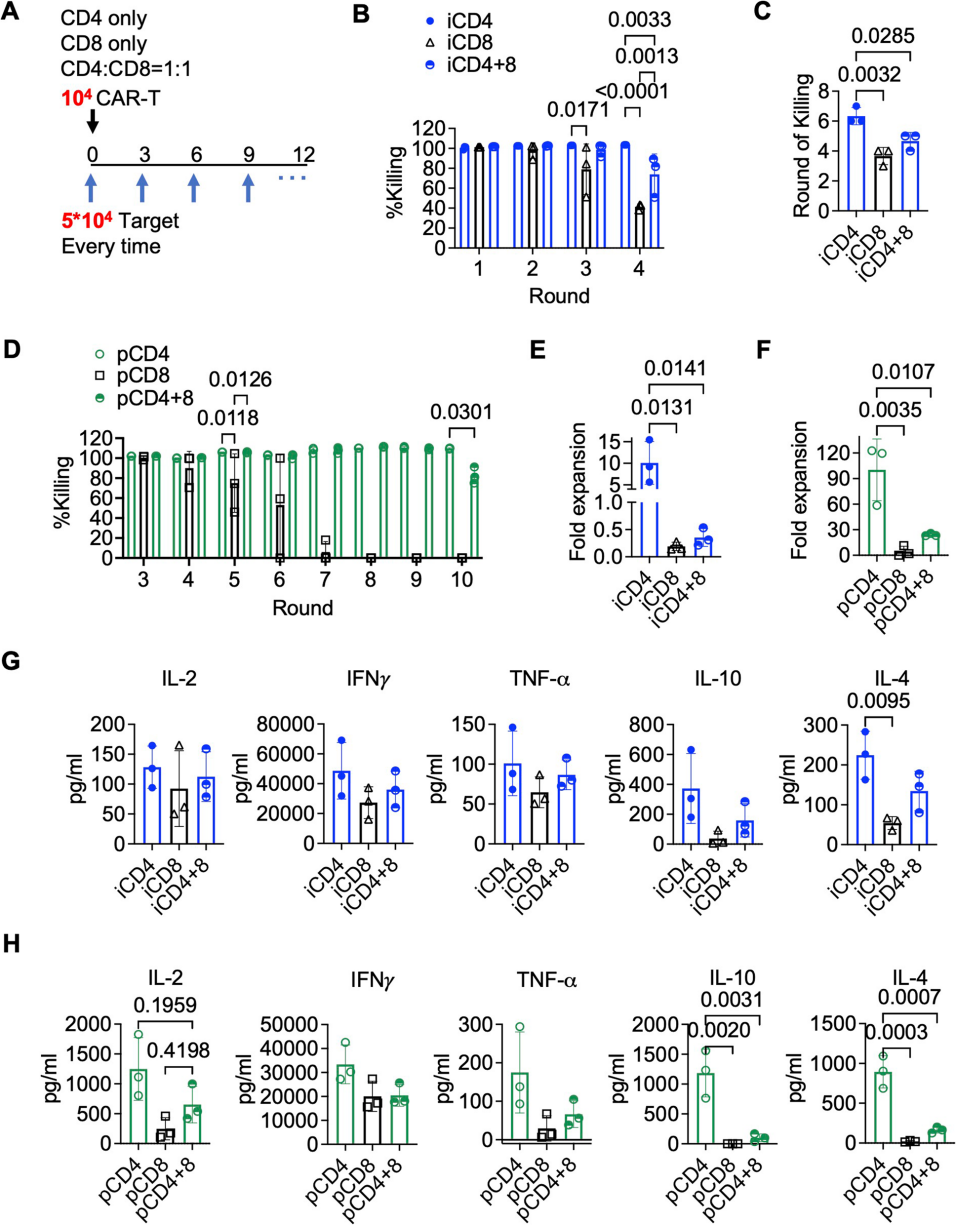
iCD4⁺ T cells exhibit robust and independent serial killing capacity. **A** Schematic illustration of the serial tumor-killing assay. **B** Serial killing efficiency across four consecutive rounds by CAR-iCD4⁺, iCD8⁺, and mixed iCD4⁺ and iCD8⁺ T cells. *n* = 3; ordinary two-way ANOVA followed by Tukey’s multiple comparisons test with a single pooled variance. **C** Maximum number of tumor-killing rounds achieved by CAR-iCD4⁺, iCD8⁺, and mixed iCD4⁺ and iCD8⁺ T cells before failing to control the growth of tumor. n = 3; ordinary one-way ANOVA followed by Tukey’s multiple comparisons test with a single pooled variance. **D** Killing efficiency from round 3 to round 10 of a 10-round serial killing assay using primary CAR-CD4⁺ and CD8⁺ T cells. *n* = 3; ordinary two-way ANOVA followed by Tukey’s multiple comparisons test with a single pooled variance. **E**, **F** Fold expansion of iPSC-derived (**E**) and primary (**F**) CAR-CD4⁺, CD8⁺, and mixed CD4⁺/CD8⁺ T cells after four rounds of serial tumor killing. *n* = 3; ordinary one-way ANOVA followed by Tukey’s multiple comparisons test with a single pooled variance. (G, H) Cytokine concentrations in culture supernatants after co-culture of NALM6 cells with iPSC-derived (**G**) or primary (**H**) CAR-CD4⁺, CD8⁺, and mixed CD4⁺/CD8⁺ T cells. n = 3; ordinary one-way ANOVA followed by Tukey’s multiple comparisons test with a single pooled variance. Note: Experiments were performed using the TKT3V1-7 iPSC line. Data are shown as mean ± SD from independent experiments (*n *values specified in figure legends) unless otherwise indicated

While short-term co-culture assays are informative for evaluating acute cytotoxic capacity, they often do not necessarily reflect long-term functional performance. Previous studies have shown that although CAR-engineered CD4⁺ T cells exhibit weaker immediate cytotoxic activity than CD8⁺ T cells, they can persist longer in patients and mediate more durable antitumor responses [10, 21, 22, 54]. To investigate the long-term functional capacity of iPSC-derived CD4⁺ and CD8⁺ T cells—and to assess whether their combination enhances therapeutic efficacy—we employed a multi-round tumor challenge assay. In this model, CAR-iCD4⁺, CAR-iCD8⁺, or 1:1 CAR-CD4⁺/CD8⁺ mixtures (1 × 10^4^ total T cells) were co-cultured with CD19⁺ NALM6 (5 × 10^4^) and re-challenged with fresh targets every 3 days. Tumor clearance was assessed after each round, with > 80% killing efficiency defined as effective tumor control (Fig. 4A). Remarkably, iCD4⁺ T cells maintained potent cytotoxicity across four consecutive challenges, consistently eliminating target cells with nearly complete efficiency. This was accompanied by sustained proliferation and robust cytokine production—including IL-2, IFN-γ, TNF-α, IL-10, and IL-4 (Fig. 4B, E, G). Effective tumor control was maintained for up to about six rounds, after which cytotoxic capacity declined (Fig. 4C). By contrast, iCD8⁺ T cells exhibited stronger initial cytotoxicity but rapidly lost killing function after three rounds and were completely ineffective following the fourth re-challenge (Fig. 4B). This functional exhaustion was accompanied by poor expansion and lower cytokine production compared to iCD4⁺ T cells (Fig. 4E, G). Primary CAR-CD4⁺ and CD8⁺ T cells followed similar trends but outperformed their iPSC-derived counterparts in terms of proliferation and sustained cytotoxicity (Fig. 4D, F). While cytokine production by primary CD8⁺ T cells resembled that of iCD8⁺ cells, primary CD4⁺ T cells secreted substantially higher levels of IL-2, IL-10, and IL-4 than iCD4⁺ T cells. Notably, IFN-γ production was comparable or even slightly higher in iCD4⁺ T cells compared to primary CD4⁺ T cells (Fig. 4G, H). IFN-γ is considered a major effector molecule mediating CAR-CD4⁺ T cell antitumor function [55]; however, a lower IL-2 level coupled with elevated IFN-γ expression may also reflect progression toward a terminal effector state. Importantly, combining CD4⁺ and CD8⁺ T cells at a 1:1 ratio—whether iPSC-derived or primary—resulted in intermediate levels of tumor control, T cell proliferation, and cytokine production, without surpassing the performance of the CD4⁺ T cell alone. In both TKT3V1-7 (Fig. 4B, C)- and FFI01s04 (Fig. S5A, B)-derived T cells, CAR-iCD4⁺ T cells alone exhibited superior serial killing capacity compared to iCD8⁺ T cells alone or CD4⁺/CD8⁺ mixtures. Together, these findings demonstrate that iPSC-derived CD4⁺ T cells are capable of mediating potent and durable antitumor responses independently of CD8⁺ T cells, underscoring their therapeutic potential as a standalone cell product.

### iCD4⁺ T cells are more resistant to exhaustion under repeated antigen stimulation

**Fig. 5:**
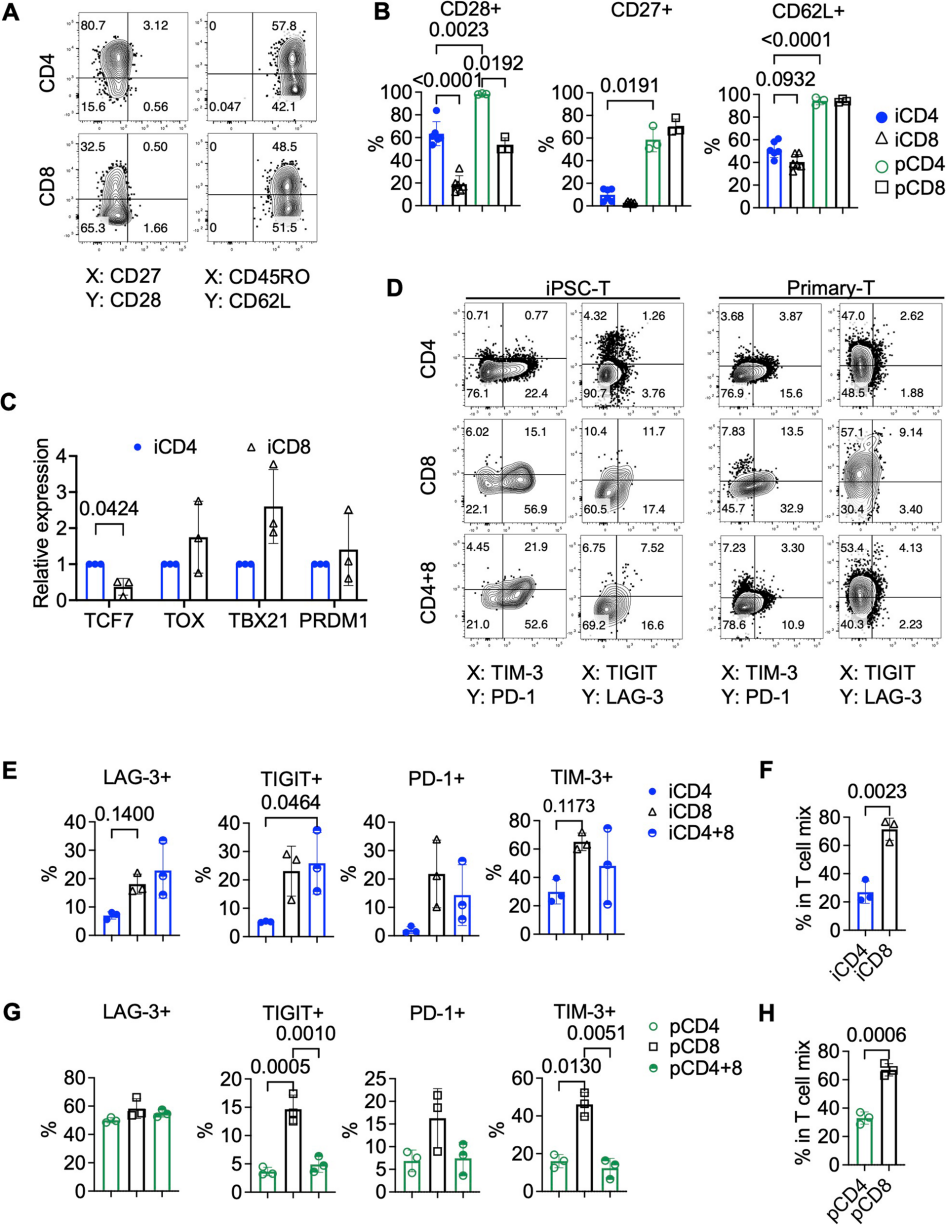
iCD4⁺ T cells maintain a memory-like phenotype and resist exhaustion. **A** Representative flow cytometry plots showing the expression of memory-associated markers on iPSC-derived CD4⁺ and CD8⁺ T cells after primary expansion. **B** Frequencies of CD28⁺, CD27⁺, and CD62L⁺ cells among primary and iPSC-derived CD4⁺ and CD8⁺ T cells following primary expansion. *n* = 6 for iPSC-derived cells; *n* = 3 for primary cells. Statistical analysis was performed using Brown-Forsythe and Welch ANOVA tests followed by Dunnett’s T3 multiple comparisons test with individual variances computed for each comparison. **C** Relative mRNA expression levels of the memory-associated transcription factor *TCF7* and exhaustion-related transcription factors *TOX*, *TBX21*, and *PRDM1* in iPSC-derived CD4⁺ and CD8⁺ T cells, assessed by quantitative PCR (qPCR). *n* = 3; paired *t* test for each gene. False discovery rate was controlled using the two-stage linear step-up procedure of Benjamini, Krieger, and Yekutieli. **D**, **E**, **G** Representative flow cytometry plots (**D**) and a summary of the expression of exhaustion markers LAG-3, TIGIT, PD-1, and TIM-3 in iPSC-derived (**E**) and primary (**G**) CAR-CD4⁺, CD8⁺, and mixed CD4⁺ and CD8⁺ T cells following four rounds of tumor killing. *n* = 3; ordinary one-way ANOVA followed by Tukey’s multiple comparisons test with a single pooled variance. **F**, **H** Proportions of CD4⁺ and CD8⁺ T cells within the CAR-T cell population at the fourth round of serial killing against NALM6 cells for iPSC-derived (**F**) and primary (**H**) T cells. *n* = 3; unpaired *t* test. Note: Experiments were performed using the TKT3V1-7 iPSC line. Data are shown as mean ± SD from independent experiments (*n* values specified in figure legends) unless otherwise indicated

T cell exhaustion is a critical obstacle to achieving durable antitumor immunity. Previous studies have suggested that the prolonged functionality of primary CD4⁺ T cells in tumor settings is partly due to their ability to maintain a less differentiated, memory-like state and resist exhaustion [10]. To assess whether iPSC-derived T cells exhibit similar resilience, we compared the memory and exhaustion profiles of iCD4⁺ and iCD8⁺ T cells before and after repeated tumor antigen stimulation. Despite their robust proliferative capacity at initial expansion (Fig. 1D), iCD4⁺ T cells retained high expression of memory-associated markers following expansion. Specifically, CD28 expression was largely preserved (Fig. 5A, B; Fig. S2A, B), and iCD4⁺ T cells showed higher levels of CD62L and co-expression of CD45RO, indicative of a central memory–like phenotype. Compared with primary T cells, iPSC-derived T cells expressed lower levels of memory markers such as CD27, CD28, and CD62L, indicating that primary T cells retain a younger and more early-memory–like phenotype. The expression level of the early memory marker CCR7 did not significantly differ between iPSC-derived CD4^+^ and CD8^+^ T cells after one round of expansion (Fig. S2E). In contrast, primary CD8^+^ T cells displayed a lower level of CCR7 expression compared with primary CD4^+^ T cells. To further dissect their differentiation state, we analyzed the expression of key transcription factors by qPCR. iCD4⁺ T cells expressed significantly higher levels of *TCF7*, which is associated with memory and stem-like phenotypes, while *TOX*, *TBX21* (T-bet), and *PRDM1* (BLIMP-1), which are associated with short-lived effector cell (SLEC) differentiation and exhaustion, were expressed at lower levels compared to iCD8⁺ T cells (Fig. 5C) [56]. These results suggest that after initial expansion, iCD8⁺ T cells are more prone to effector differentiation, whereas iCD4⁺ T cells are biased toward memory maintenance.

Before challenge with tumor cells, the expression of exhaustion-associated surface markers—including PD-1, TIM-3, LAG-3, TIGIT, and CTLA-4—was comparably low between iCD4⁺ and iCD8⁺ T cells (Fig. S2H). Then, following multiple rounds of tumor challenge, we assessed T cell exhaustion by monitoring the expression of inhibitory receptors. iCD8⁺ T cells showed increased expression of exhaustion markers such as LAG-3, TIGIT, PD-1, and TIM-3, consistent with the development of a dysfunctional state (Fig. 5D, E). In contrast, iCD4⁺ T cells maintained low expression of these markers even after repeated stimulation. In the OKT3 repetitive stimulation model (Fig. S7A), both iPSC-derived CD4⁺ and CD8⁺ T cells progressively lost IL-2 production after repeated stimulation. Notably, iCD8⁺ T cells exhibited a substantial decline in IFN-γ and TNF-α production upon the second stimulation, whereas iCD4⁺ T cells largely preserved their IFN-γ and TNF-α secretion capacity (Fig. S7B, C), suggesting a higher resistance to exhaustion. Primary CD4⁺ and CD8⁺ T cells showed similar subset-specific trends, further reinforcing the notion that CD4⁺ T cells are intrinsically less prone to exhaustion (Fig. 5G). These differences in exhaustion kinetics, proliferation, and long-term cytotoxicity between iCD4⁺ and iCD8⁺ T cells parallel those observed in primary T cell subsets. Whereas CD8⁺ T cells preferentially differentiate into SLECs that mediate rapid but transient effector responses, CD4⁺ T cells—including those derived from iPSCs—exhibit more durable yet moderate functional profiles. Notably, co-culturing CAR-iCD4⁺ and CAR-iCD8⁺ T cells at a 1:1 ratio did not mitigate their exhaustion phenotype (Fig. 5D, E). In contrast, the same 1:1 mixture of primary CD4⁺ and CD8⁺ T cells resulted in reduced exhaustion marker expression after repeated tumor challenge. Given that CD8⁺ T cells remained the dominant population in both settings (Fig. 5F, H), this difference likely reflects superior helper function in primary CD4⁺ T cells compared to their iPSC-derived counterparts.

### Providing help to iCD8⁺ T cells constrains the functional state of iCD4⁺ T cells themselves

**Fig. 6:**
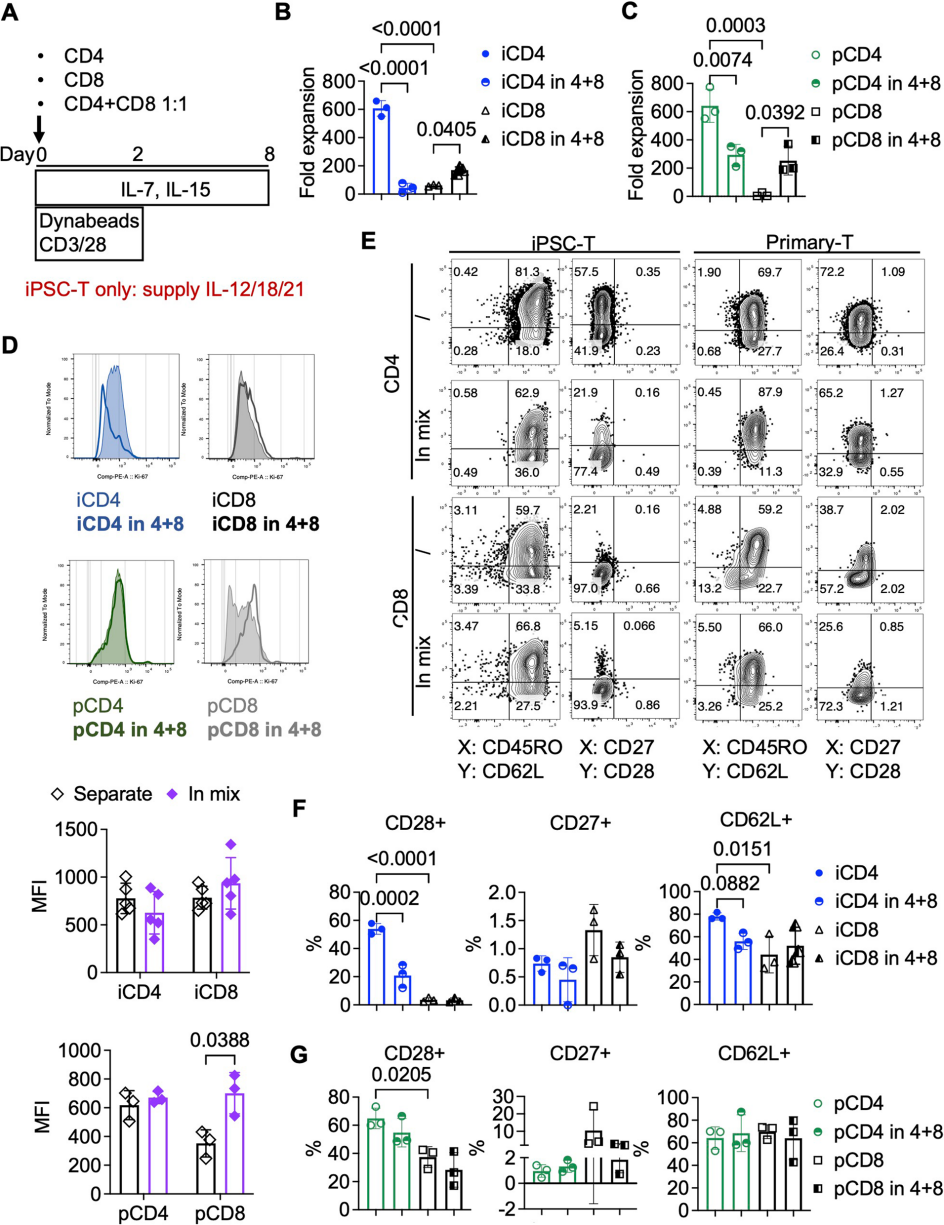
iCD4⁺ T cells provide helper support to iCD8⁺ T cells. **A** Schematic illustration of co-expansion of CD4⁺ and CD8⁺ T cells. **B**, **C** Fold expansion over 8 days of iPSC-derived (**B**) and primary (**C**) CD4⁺ and CD8⁺ T cells cultured alone or in a 1:1 CD4⁺/CD8⁺ mix. *n* = 3; RM one-way ANOVA followed by Tukey’s multiple comparisons test with a single pooled variance. **D** Representative flow cytometry plots showing Ki-67 expression in iPSC-derived and primary CD4⁺ and CD8⁺ T cells on day 8 after stimulation, when cultured alone or in a 1:1 CD4⁺/CD8⁺ mix. The lower panel shows the summarized results from five iPSC-T cell lots and three primary T cell lots. *n* = 5 (iPSC-T), *n* = 3 (primary T). Statistical analysis was performed using RM two-way ANOVA with matched values across rows, followed by Šídák’s multiple comparisons test with a single pooled variance. **E**–**G** Representative flow cytometry plots (**E**) and summary of the frequencies of CD28⁺, CD27⁺, and CD62L⁺ cells among iPSC-derived (**F**) and primary (**G**) CD4⁺ and CD8⁺ T cells on day 8 of stimulation, cultured alone or in a 1:1 CD4⁺/CD8⁺ mix. *n* = 3; RM one-way ANOVA followed by Tukey’s multiple comparisons test with a single pooled variance. Note: Experiments were performed using the TKT3V1-7 iPSC line. Data are shown as mean ± SD from independent experiments (*n* values specified in figure legends) unless otherwise indicated

CD4⁺ T cells are well known to support CD8⁺ T cell function, as demonstrated in numerous studies on chronic infection [57, 58] and cancer [59]. While our multi-round tumor challenge assay did not reveal a clear enhancement of CAR-iCD8⁺ T cell function by CAR-iCD4⁺ T cells, the consistent dominance of iCD8⁺ T cells in the CD4/CD8 co-culture group suggests functional interplay between the two subsets (Fig. 5F, H) [10]. To directly evaluate whether iCD4⁺ T cells provide helper signals to iCD8⁺ T cells, we assessed CD8⁺ T cell proliferation and memory phenotype in the presence or absence of CD4⁺ T cells (Fig. 6A). CD4⁺ and CD8⁺ T cells—derived from either iPSCs or primary sources—were mixed at a 1:1 ratio and expanded as described above. We found that CD4⁺ T cells enhanced the proliferation of CD8⁺ T cells in both primary and iPSC settings. However, this helper effect came at a significant cost: CD4⁺ T cell proliferation was markedly impaired under the same conditions (Fig. 6B, C). This suppression was not due to increased apoptosis, as no signs of significant cell death were observed under the microscope. Instead, Ki-67 expression decreased in iCD4⁺ T cells and increased in iCD8⁺ T cells in the mixed culture, indicating that the shift in fold expansion was driven primarily by differential proliferation rather than survival (Fig. 6D). While primary CD4⁺ T cells also exhibited reduced proliferation when co-cultured with CD8⁺ T cells, the effect was less pronounced, and Ki-67 levels declined only modestly (Fig. 6C, D). Given the strongly pro-inflammatory nature of the culture conditions and the lack of IL-10 production by CD8⁺ T cells (Fig. 4G, H), the presence of CD8⁺ regulatory T cells is unlikely. Instead, we propose that CD8⁺ T cells—due to their limited intrinsic cytokine production—may heavily rely on CD4⁺ T cell–derived cytokines. This competition for shared cytokines likely suppresses CD4⁺ T cell proliferation, especially in iCD4⁺ T cells, which secrete lower levels of IL-2 and IL-21 compared to their primary counterparts (Figs. 2A, 4G, H) [58, 60, 61]. Phenotypic analysis revealed no increase in memory-associated marker expression in CD8⁺ T cells as a result of CD4⁺ T cell presence. In contrast, CD4⁺ T cells—both primary and iPSC-derived—exhibited downregulation of CD28, and iCD4^+^ T cell also downregulated CD62L, suggesting a loss of central memory–like features (Fig. 6F, G). These findings are in line with a previous report by D. Wang et al., which showed that CD8⁺ T cells can negatively influence the memory phenotype and function of CD4⁺ T cells [10]. In conclusion, while iCD4⁺ T cells can provide limited direct helper effects to iCD8⁺ T cells, this support comes at the expense of their own proliferative capacity and memory maintenance. These observations underscore the importance of optimizing co-culture strategies and cytokine conditions when using iCD4⁺ T cells therapeutically, in order to preserve their functional integrity.

## Discussion

In this research, we investigated the therapeutic potential of CD4⁺ T cells in the context of iPSC-based T cell immunotherapy, an area of growing interest in recent years. While previous studies have shown that ATO-derived iPSC-CD4⁺ T cells possess the capacity to differentiate into regulatory T cells [28], our findings demonstrate that, under type 1 cytokine stimulation, these cells can also acquire cytotoxic effector functions. We observed that iPSC-derived CD4⁺ and CD8⁺ T cells recapitulate many of the hallmark functional differences seen between their primary counterparts. These can be summarized into two principal aspects: first, under type 1 expansion conditions, CD4⁺ T cells adopt a dual phenotype characterized by moderate cytotoxic activity coupled with helper functionality, whereas CD8⁺ T cells exhibit a more potent cytotoxic profile along with increased expression of NK cell–associated features [15]. Second, CD4⁺ T cells are more proficient at maintaining a memory-like phenotype, displaying greater resistance to exhaustion and improved tolerance to repeated antigen stimulation. In contrast, CD8⁺ T cells tend to mount robust but transient effector responses and are more susceptible to functional decline in the context of chronic antigen exposure [10, 22].

T cell exhaustion is a major barrier to effective tumor immunotherapy [62, 63], with most mechanistic studies focusing on CD8⁺ T cells, while CD4⁺ T cell exhaustion is comparatively underexplored. Although it has been proposed that CD4⁺ T cells resist exhaustion due to more tightly regulated proliferation [64], our findings show that iCD4⁺ T cells exhibit greater proliferative capacity than iCD8⁺ T cells, yet remain more resistant to exhaustion. This suggests the presence of proliferation-independent mechanisms. D. Wang et al. previously proposed that enhanced activation of the Wnt signaling pathway may underlie the superior exhaustion resistance of primary CAR-CD4⁺ T cells, given the well-established role of Wnt signaling in promoting memory T cell formation [10, 65]. Consistent with this notion, reanalysis of published RNA-seq data revealed that ATO-derived ESC-CD4⁺ T cells are enriched in the Wnt signaling pathway (Fig. S6). ESC-CD4⁺ T cells also exhibited enrichment in homeostasis-associated pathways, while ESC-CD8⁺ T cells were skewed toward activation-related programs such as MAPK signaling, calcium-mediated signaling, leukocyte activation, and NF-κB signaling (Fig. S6). Excessive activation has been linked to poor persistence and memory formation [66–71], implying that the restrained activation profile of iCD4⁺ T cells may support their durability. Moreover, iCD4⁺ T cells secrete IL-2, IL-21, and upregulate CD40L upon activation, potentially sustaining their own function and supporting surrounding immune cells through autocrine and paracrine effects [17, 72–75].

The therapeutic synergy between CAR-CD4⁺ and CAR-CD8⁺ T cells remains a subject of ongoing debate [10, 76]. For instance, C.J. Turtle et al. reported that a 1:1 mixture of CAR-CD4⁺ and CD8⁺ T cells was more effective than either subset alone, a finding supported by clinical trials treating adult B cell ALL patients [20, 76]. In contrast, studies by D. Wang et al. and Y. Yang et al. demonstrated that CAR-CD4⁺ T cells alone outperformed both CD8^+^ T cells and mixed products in models of glioblastoma and leukemia [10, 22]. In our ALL model utilizing CD19-BBζ CAR-T cells—both primary and iPSC-derived—we observed that the 1:1 CD4/CD8 mixed group exhibited intermediate antitumor efficacy: superior to CD8⁺ T cells alone but less effective than CD4⁺ T cells alone. This was not simply additive: CD4⁺ T cells significantly enhanced CD8⁺ T cell expansion, while their own proliferation and memory phenotype were suppressed—both in primary and iPSC-derived settings [10]. These findings support a direct helper role for iPSC-derived CD4⁺ T cells with a trade-off: their own functional integrity may be compromised within the tumor milieu. Although our culture conditions were highly type 1 biased—rendering the induction of regulatory CD8⁺ T cells unlikely—and both iPSC- and primary-derived CD8⁺ T cells expressed minimal IL-10 [77–80], competition for key cytokines such as IL-2 and IL-21, or interference with CD40L signaling, may underlie the impaired proliferation of CD4⁺ T cells [17–19, 52, 61]. These outcomes may vary with CAR constructs and tumor types [81], but since iCD4⁺ T cells faithfully recapitulate many functional features of primary CD4⁺ T cells, it is reasonable to expect that iCD4⁺ T cells could be effective in tumor settings where primary CD4⁺ T cell–based therapies have shown clinical benefit.

This study underscores the therapeutic potential of iCD4⁺ T cells in cancer immunotherapy; however, several critical challenges remain before clinical translation can be achieved. On one hand, the in vitro yield of iCD4⁺ T cells remains relatively low, and the ATO-based differentiation protocol lacks precise control over CD4⁺ versus CD8⁺ lineage commitment. Additionally, the use of murine MS5 stromal feeder cells introduces xenogeneic elements, complicating manufacturing scalability and regulatory compliance [26]. On the other hand, iCD4⁺ T cells differ from their primary counterparts in both development and function. MS5 feeder cells lack the full range of positive and negative selection signals essential for proper T cell maturation, and the hematopoietic progenitor cells derived from EB cultures resemble fetal rather than adult hematopoiesis [26]. iCD4⁺ T cells still exhibit innate-like characteristics and reduced durability in cytotoxicity, cytokine secretion, and proliferation. They also show less upregulation of inhibitory receptors such as PD-1 and LAG-3 compared to primary cells, suggesting differences in activation signaling strength or regulatory thresholds [42]. To enhance their therapeutic efficacy, targeted enhancements such as CAR optimization, cytokine armoring, or combination with checkpoint blockade (e.g., anti–PD-L1 [82]) may be needed. Furthermore, the indirect antitumor function of CD4^+^ T cells, such as facilitating antigen cross-priming via antigen-presenting cells and activating the host immune system [15, 83–85] remains underexplored in the iPSC context due to challenges in TCR–MHC matching. Developing appropriate preclinical models will be essential. We are actively investigating the humanized mouse model to conduct subsequent in vivo functional validation experiments. Finally, the safety profile of iCD4⁺ CAR-T cells, particularly regarding risks like cytokine release syndrome [54], must be carefully evaluated in future studies.

This study primarily focused on the functional attributes of iCD4⁺ T cells relevant to therapeutic application. Accordingly, we employed a type 1–skewed cytokine environment during in vitro expansion and did not explore the potential of iCD4⁺ T cells to differentiate toward type 2, type 3, or T follicular helper (Tfh) lineages. This decision was based not only on the limited translational relevance of those subsets for current immunotherapy strategies, but also on practical constraints: iCD4⁺ T cells exhibited poor expansion in the absence of IL-12, IL-18, and IL-21, making alternative differentiation protocols technically challenging. While the functionality of in vitro–differentiated T cells often falls short of that observed in cells generated through physiological development, the iPSC system and in vitro platforms offer unique advantages. Notably, they provide a powerful model to dissect how developmental cues shape long-term T cell functionality, including resistance to exhaustion. For example, the strength and duration of TCR signaling, as well as auxiliary pathways, may play critical roles at specific developmental windows in establishing the exhaustion-resistant phenotype of CD4⁺ T cells. Harnessing the controlled environment of in vitro differentiation could thus offer valuable insights into the ontogeny of durable and functionally resilient T cell lineages.

## Conclusions

This study demonstrates that iPSC-derived CD4⁺ T cells exhibit both helper and cytotoxic properties, closely mirroring the functional dichotomy of their primary counterparts. Importantly, it is the first report demonstrating that iCD4^+^ T cells integrate proliferative capacity, resistance to exhaustion, and the ability to support CD8⁺ T cell responses, thereby offering a unique therapeutic profile distinct from iCD8⁺ T cells. These findings highlight the relevance of iCD4⁺ T cells as a renewable, off-the-shelf source for CAR-T immunotherapy and underscore their potential to overcome key barriers such as persistence and functional decline. Beyond their translational implications, iPSC-based systems provide a powerful model to dissect the developmental cues shaping T cell fate and durability, opening new avenues for rational engineering of next-generation immunotherapies.

## Supplementary Information

Supplementary Material 1: Figure S1. Differentiation of various iPSC lines into T cells using the artificial thymic organoid (ATO) system. Figure S2. Phenotypic analysis of iPSC-derived CD4⁺ and CD8⁺ T cells before and after initial expansion. Figure S3. Activation phenotype of iCD4⁺ and iCD8⁺ T cells. Figure S4. Cytokine production capacity of iCD4⁺ and iCD8⁺ T cells. Figure S5. Functional characterization of αβ CD4⁺ T cells derived from non-T iPSC line FFI01s04 (FF-iCD4.^+^ T Cells). Figure S6. Functional enrichment analysis of differentially expressed genes between ATO-derived ESC-CD4⁺ and ESC-CD8⁺ T cells. Figure S7. Cytokine production by iPSC-derived CD4⁺ and CD8⁺ T cells during serial stimulation.

## Data Availability

All data generated or analyzed during this study is included in this published article and its supplementary information files.

## Abbreviations

iPSC: Induced pluripotent stem cell
ATO: Artificial thymic organoid
ALL: Acute lymphoblastic leukemia
TCR: T cell receptor
CAR: Chimeric antigen receptor
Treg: Regulatory T cell
PBMC: Peripheral blood mononuclear cells
DLL4: Delta-like ligand 4
EB: Embryoid body
HPC: Hematopoietic progenitor cell
scFv: Single-chain variable fragment
FLuc: Firefly luciferase
CBA: Cytometric bead array
SP: Single positive cell
ILC: Innate lymphoid cell
SLEC: Short-lived effector cell
