# Supplementary material for: Adaptive-like CAR-iPSC-CD4⁺ T cells outperform CD8⁺ counterparts in sustained ALL control
Source: Inflamm Regen. 2026 Jan 3;46:4. doi: 10.1186/s41232-025-00402-4 (PMC12805770; doi:10.1186/s41232-025-00402-4)
Supplement: Supplementary file 1 — Supplementary Material 1: Figure S1. Differentiation of various iPSC lines into T cells using the artificial thymic organoid (ATO) system. Figure S2. Phenotypic analysis of iPSC-derived CD4⁺ and CD8⁺ T cells before and after initial expansion. Figure S3. Activation phenotype of iCD4⁺ and iCD8⁺ T cells. Figure S4. Cytokine production capacity of iCD4⁺ and iCD8⁺ T cells. Figure S5. Functional characterization of αβ CD4⁺ T cells derived from non-T iPSC line FFI01s04 (FF-iCD4.+ T Cells). Figure S6. Functional enrichment analysis of differentially expressed genes between ATO-derived ESC-CD4⁺ and ESC-CD8⁺ T cells. Figure S7. Cytokine production by iPSC-derived CD4⁺ and CD8⁺ T cells during serial stimulation. [file 41232_2025_402_MOESM1_ESM.docx]

Supplementary Material

**Figure S1. Differentiation of various iPSC lines into T cells using the artificial thymic organoid (ATO) system**

**(A)** Representative flow cytometry plots showing the time-course of T cell differentiation from hematopoietic progenitor cells (HPCs) in ATO cultures. Cells were analyzed on days 18, 29, and 49 after initiation of ATO differentiation for expression of T cell lineage markers: CD5, CD7, CD4, CD8β, CD1a, CD3, and TCRαβ.

**(B)** CD4⁺ and CD8⁺ T cell frequencies derived from different iPSC lines following ATO differentiation. Data are represented as mean ± SD. n=11 independent experiments; Paired t test.
FF: non-T iPSC line FFI01s04;
TKT: T-iPSC line TKT3V1-7;
b3a2: T-iPSC line b3a2#9, which processing a BCR-ABL–restricted, HLA-DR9–restricted b3a2 TCR;
FF-b3a2: non-T iPSC line FFI01s04 transduced with the HLA class II–restricted b3a2 TCR.


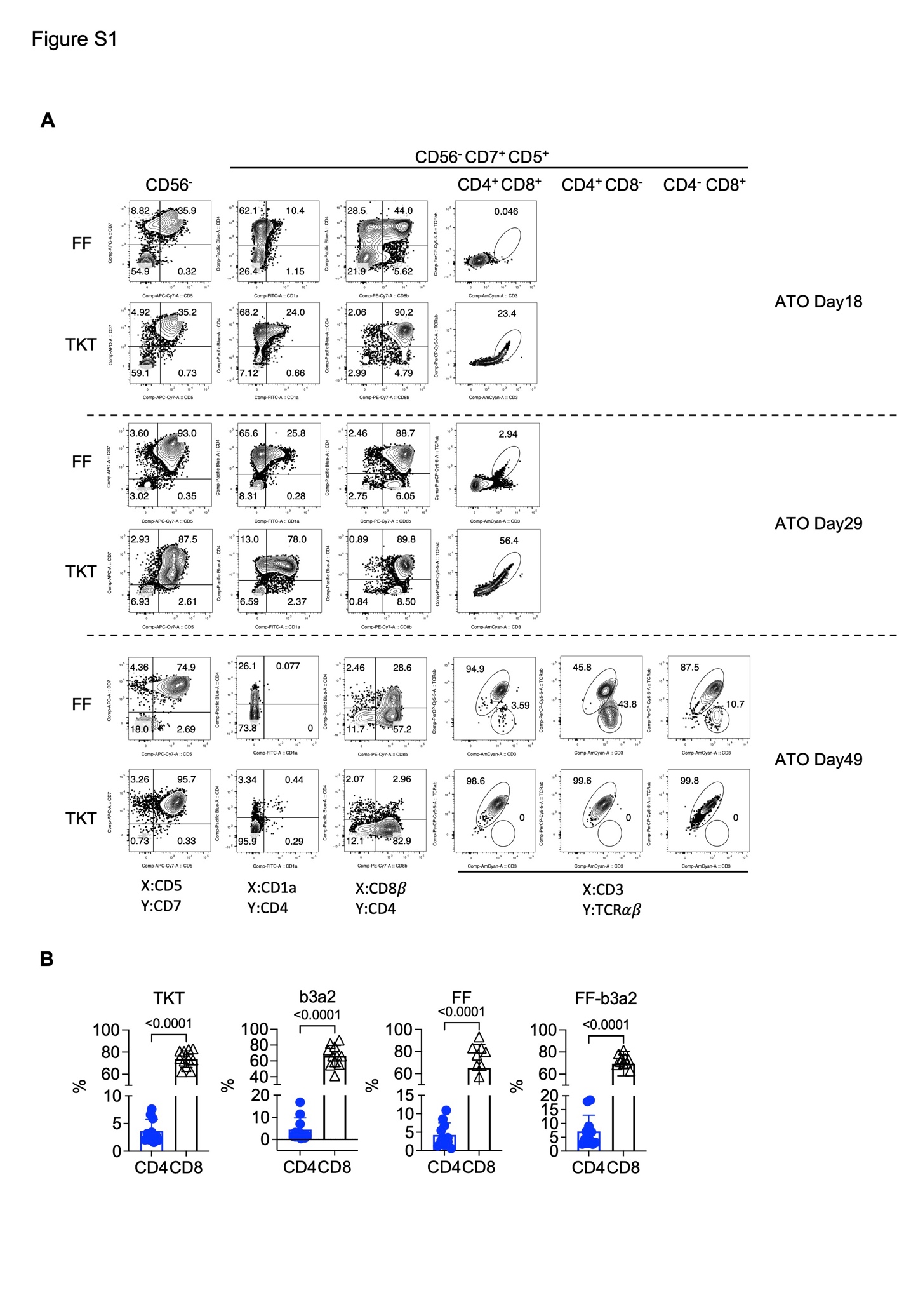


**Figure S2. Phenotypic analysis of iPSC-derived CD4⁺ and CD8⁺ T cells before and after initial expansion**

**(A)** Representative flow cytometry plots showing the phenotype of ATO-derived iCD4⁺ and iCD8⁺ T cells prior to initial expansion. Naïve CD4⁺ T cell–associated chemokine receptors and surface markers (CCR4, CCR7, CD45RA, CD28), as well as killer ligands (NKG2D, TRAIL), are shown.

**(B)** Frequencies of CD28⁺, CCR4⁺, and NKG2D⁺ cells among iPSC-derived CD4⁺ and CD8⁺ T cells. n = 4; paired t test.

**(C)** Fold expansion over 8 days of primary CD4⁺ and CD8⁺ T cells. n = 3 (donors); paired t test.

**(D)** Fold expansion over 8 days of iCD4⁺ and iCD8⁺ T cells following initial stimulation. T cells were derived from non-T iPSC line FFI01s04 and expressed TCRαβ. n = 5.

**(E)** Mean fluorescence intensity (MFI) of CCR7 in iPSC-derived and primary CD4⁺ and CD8⁺ T cells after one round of expansion. iPSC-T cells: n = 6; primary T cells: n = 3; ordinary one-way ANOVA followed by Tukey’s multiple comparisons test with a single pooled variance.

**(F)** Representative flow cytometry plots showing the expression of chemokine receptors CXCR3 and CCR4 in resting iPSC-derived and primary CD4⁺ /CD8⁺ T cells following one round of expansion.

**(G)** Expression frequencies of chemokine receptors CXCR3 and CCR4 in resting iPSC-derived and primary CD4⁺/CD8⁺ T cells following one round of expansion. iPSC-T cells: n = 6; primary T cells: n = 3. Statistical analysis was performed using Brown-Forsythe and Welch ANOVA tests followed by Dunnett’s T3 multiple comparisons test with individual variances computed for each comparison.

**(H)** Mean fluorescence intensity (MFI) of inhibitory receptors on resting iPSC-derived and primary CD4⁺ and CD8⁺ T cells after one round of expansion. n = 3; ordinary one-way ANOVA followed by Tukey’s multiple comparisons test with a single pooled variance.

Note: Experiments were performed using the TKT3V1-7 iPSC line unless otherwise specified. Data are shown as mean ± SD from independent experiments (n values specified in figure legends) unless otherwise indicated.


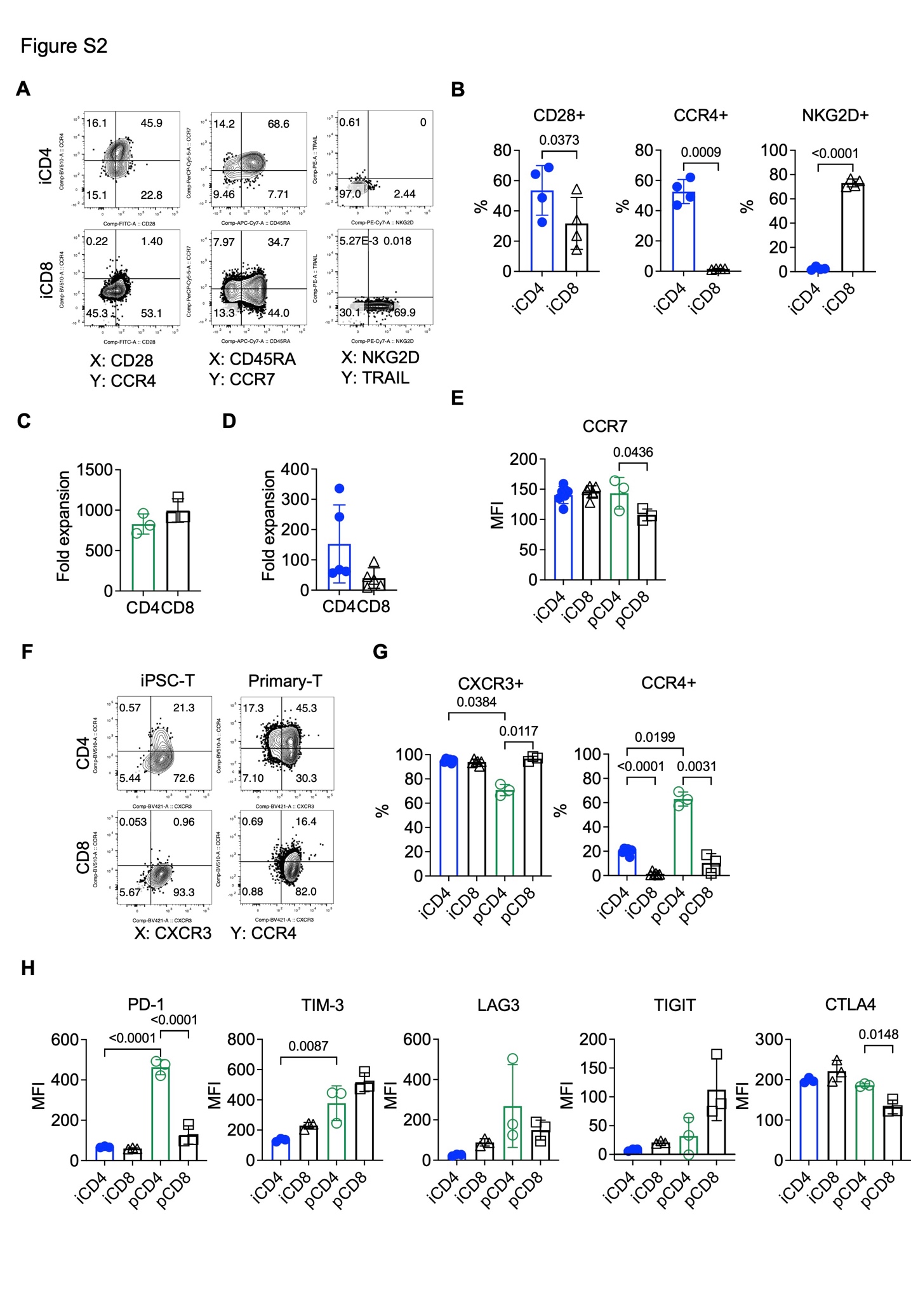


**Figure S3. Activation phenotype of iCD4⁺ and iCD8⁺ T cells**

Representative flow cytometry plots showing surface expression of activation markers, co-stimulatory receptors, and inhibitory receptors on primary and iPSC-derived CD4⁺ and CD8⁺ T cells after 24-hour stimulation with plate-bound anti-CD3 antibody OKT3 or PMA/ionomycin.
Activation markers: CD25, CD69;
Co-stimulatory receptors: CD27, CD28, 4-1BB, ICOS, CD40L;
Inhibitory receptors: PD-1, TIM-3, LAG-3, TIGIT, CTLA-4.


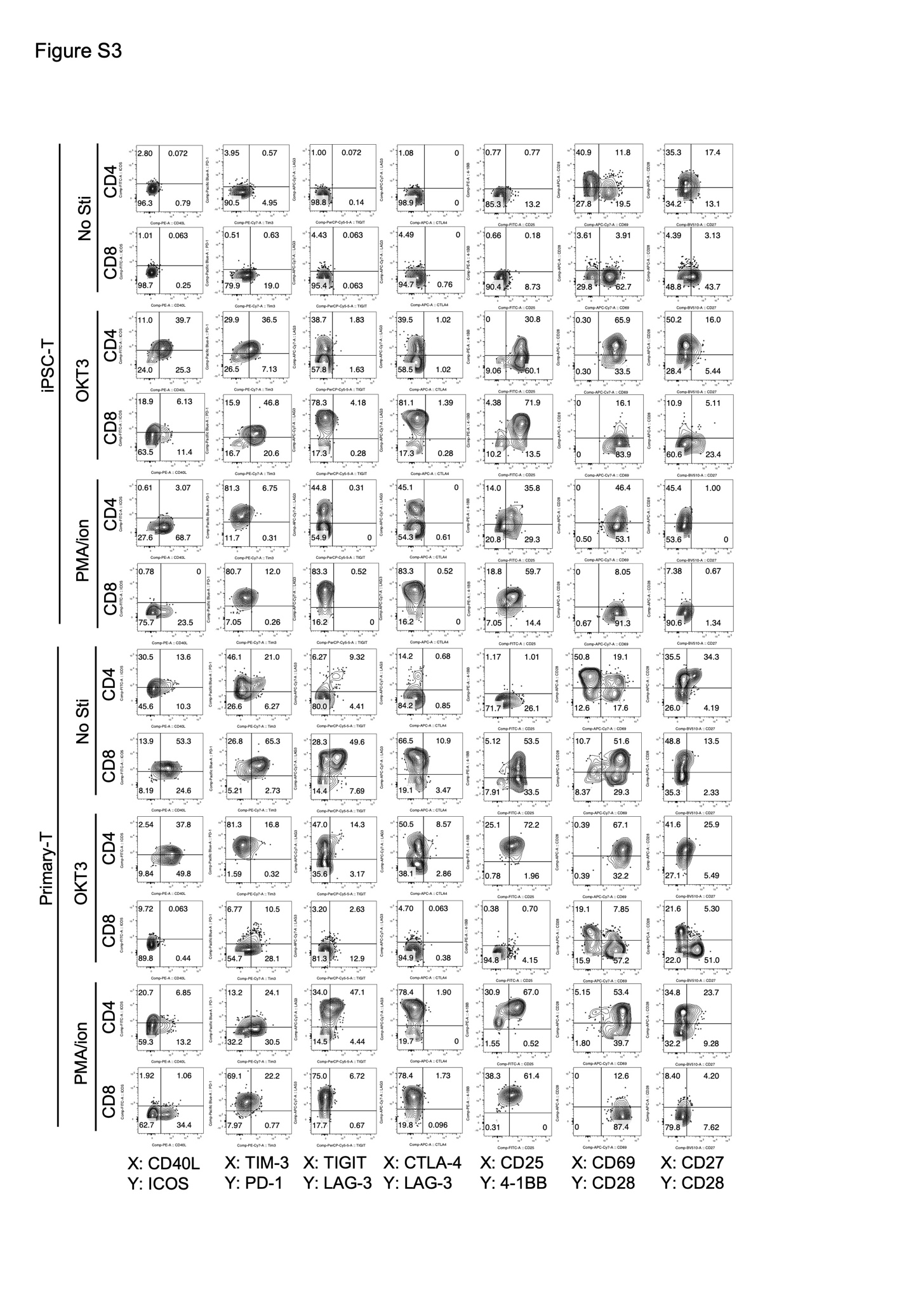


**Figure S4. Cytokine production capacity of iCD4⁺ and iCD8⁺ T cells**

**(A)** Representative flow cytometry plots showing intracellular cytokine production by iPSC TKT3V1-7-derived and primary CD4⁺ and CD8⁺ T cells following 4-hour stimulation with PMA/ionomycin in the presence of monensin.

**(B, C)** Representative flow cytometry plots **(B)** and summary **(C)** showing intracellular cytokine production by iPSC FFI01s04-derived iCD4⁺ and iCD8⁺ T cells after 4-hour stimulation with PMA/ionomycin in the presence of monensin. n = 2 independent experiments; paired t test.

Cytokines and cytotoxic molecules analyzed include IL-2, IL-4, IL-21, IFN-γ, TNF-α, and granzyme B.


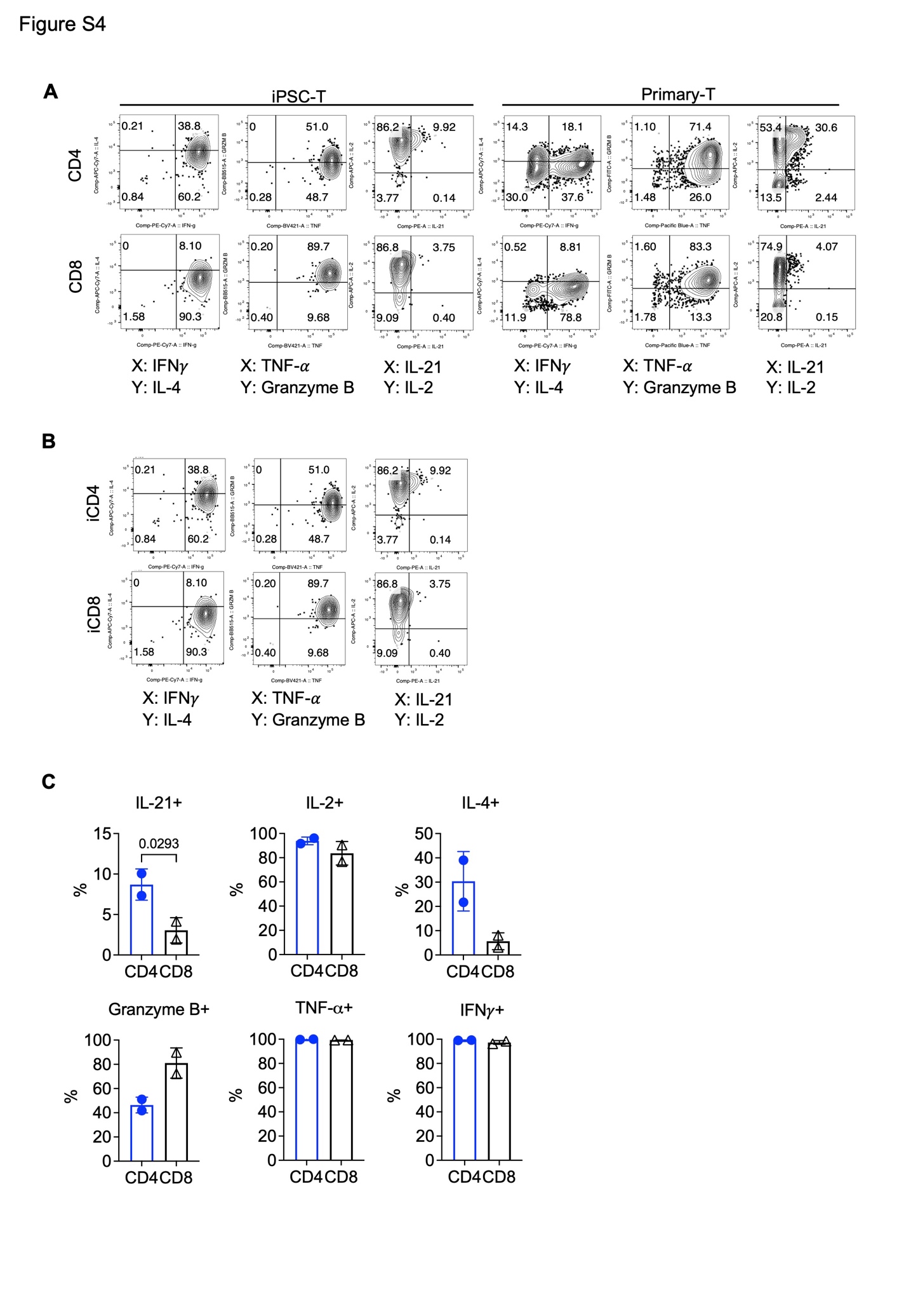


**Figure S5. Functional characterization of αβ CD4⁺ T cells derived from non-T iPSC line FFI01s04 (FF-iCD4^+^ T Cells)**

**(A)** Serial tumor-killing efficiency over four consecutive rounds by CAR-expressing FF-iCD4⁺, iCD8⁺, and CD4⁺ /CD8⁺ mixed T cells. n = 3; ordinary two-way ANOVA followed by Tukey’s multiple comparisons test with a single pooled variance.

**(B)** Maximum number of tumor-killing rounds achieved by CAR-expressing iCD4⁺, iCD8⁺, and CD4⁺ /CD8⁺ mixed T cells before loss of tumor control. n = 3.

Note: Data are shown as mean ± SD from independent experiments (n values specified in figure legends) unless otherwise indicated.

**
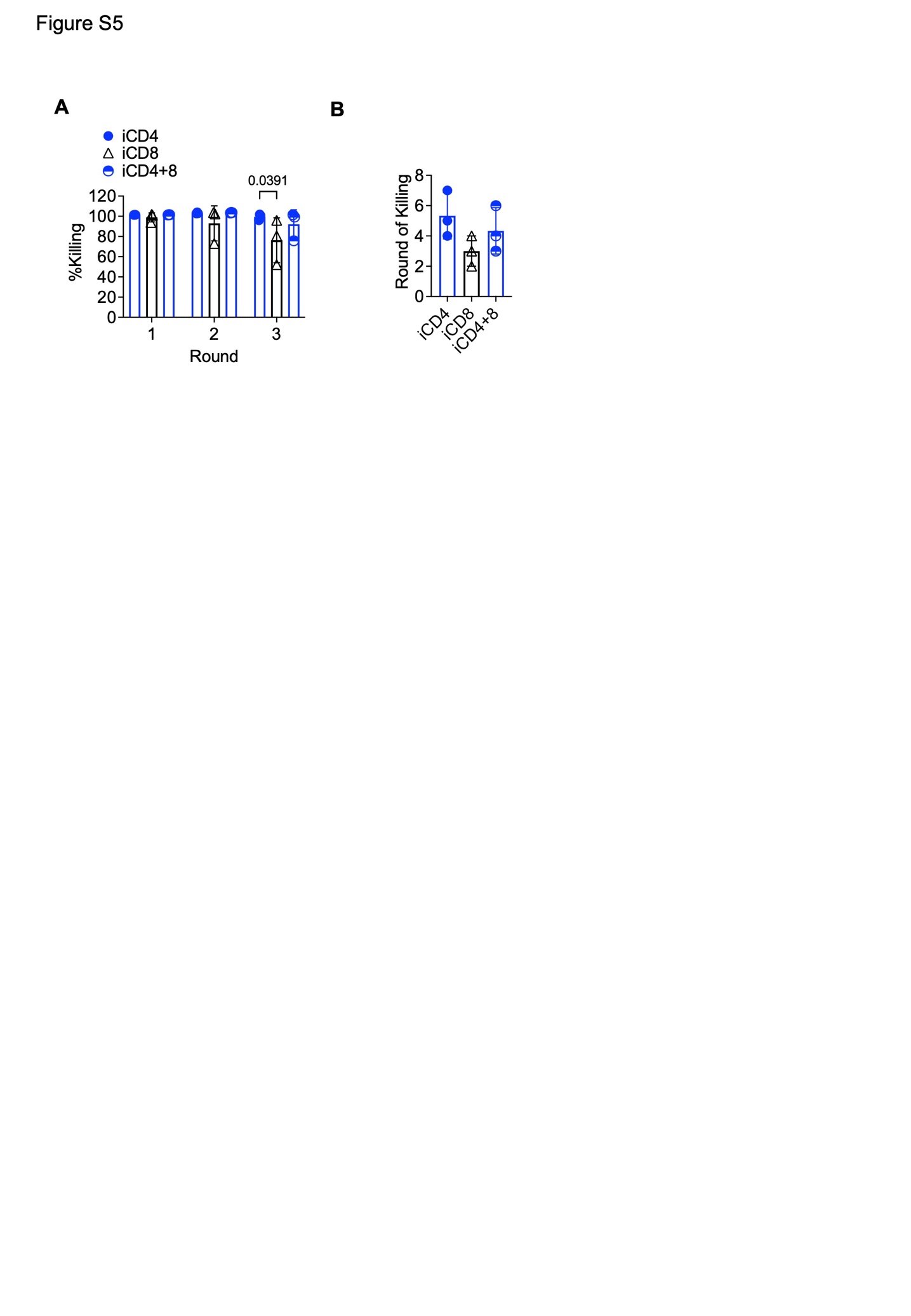
 Figure S6. Functional enrichment analysis of differentially expressed genes between ATO-derived ESC-CD4⁺ and ESC-CD8⁺ T cells**

Gene Ontology (GO) and pathway enrichment analyses of the top 250 differentially expressed genes (DEGs) in ATO-derived ESC-CD4⁺ and ESC-CD8⁺ T cells were performed using Metascape. DEGs were identified from RNA-seq data (GSE116015) using GEO2R, and enrichment analysis was conducted using Metascape ([https://metascape.org](https://metascape.org/)) (1).


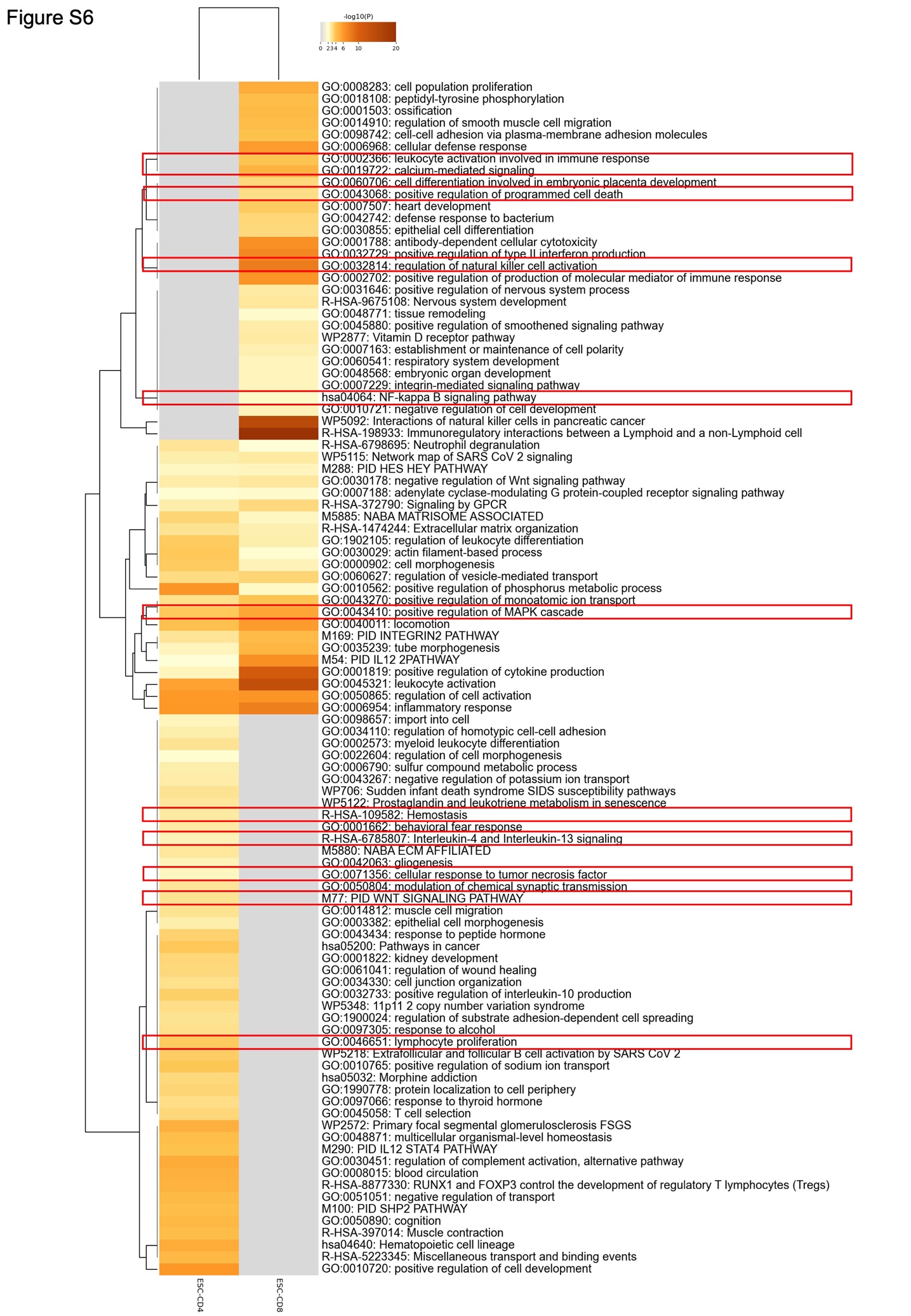


**Figure S7. Cytokine production by iPSC-derived CD4⁺ and CD8⁺ T cells during serial stimulation.**
(A) Schematic of the experimental design. Once-expanded, cryopreserved iPSC-derived CD4⁺ and CD8⁺ T cells were thawed and stimulated on immobilized OKT3–coated plates (1 μg/mL). Intracellular cytokine production was analyzed using the PMA/ionomycin/monensin assay. OKT3-coated plates were replaced every 3 days, and 5 ng/mL IL-7 was supplemented throughout the culture.
(B, C) Representative flow cytometry plots (B) and summary graphs (C) showing intracellular cytokine production (IL-2, IFN-γ, TNF-α, and Granzyme B) by iPSC-derived CD4⁺ and CD8⁺ T cells at three time points: freshly thawed (Thaw), day 3 after the first stimulation (S1), and day 3 after the second stimulation (S2). n = 3 independent experiments. Statistical analysis was performed using ordinary two-way ANOVA followed by Šídák’s multiple comparisons test with a single pooled variance.

Note: Experiments were performed using the TKT3V1-7 iPSC line unless otherwise specified. Data are shown as mean ± SD unless otherwise indicated.


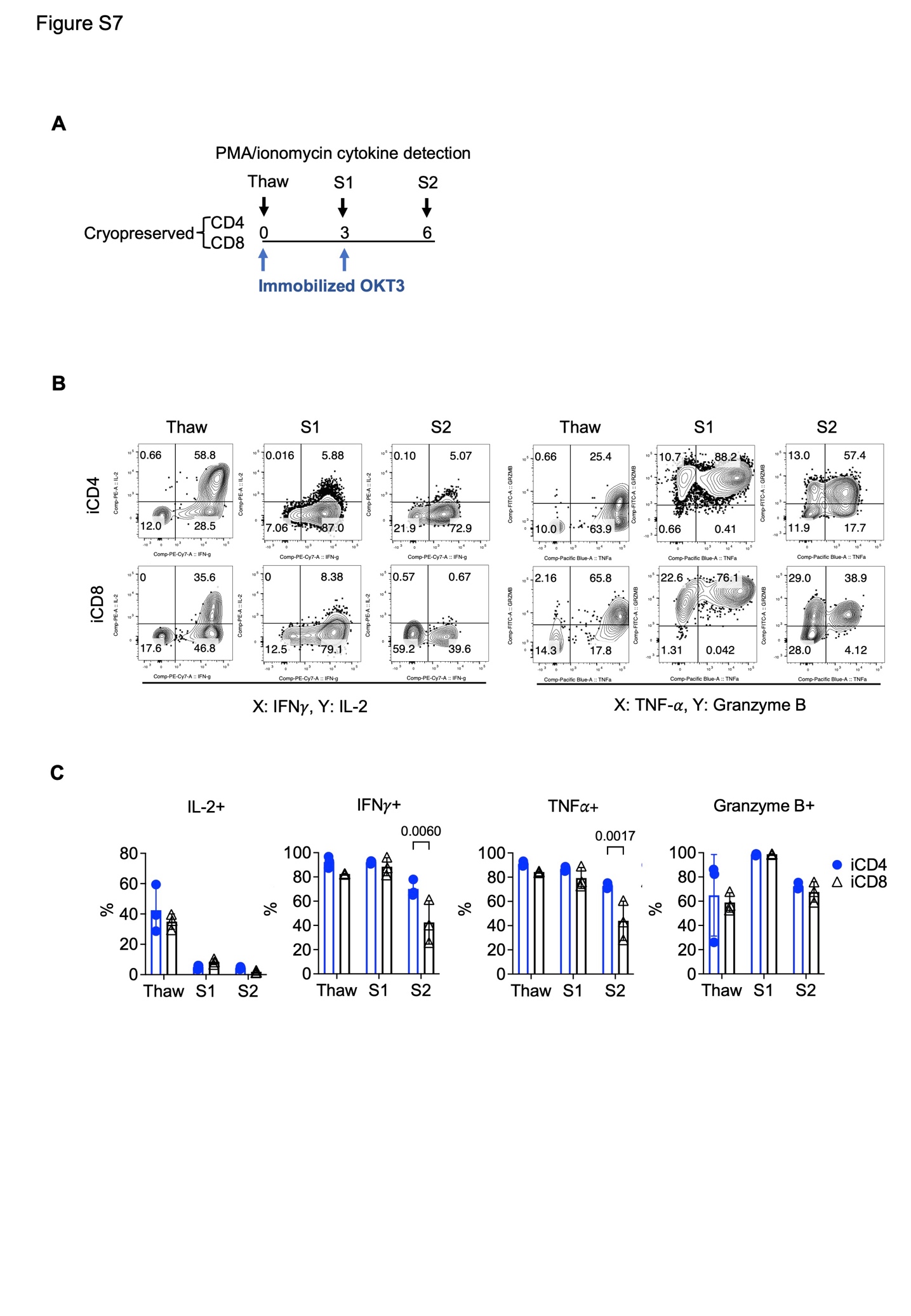


1. Montel-Hagen A, Seet CS, Li S, Chick B, Zhu Y, Chang P, et al. Organoid-Induced Differentiation of Conventional T Cells from Human Pluripotent Stem Cells. Cell Stem Cell. 2019;24(3):376-89 e8.
